# Soluble TRAIL Armed Human MSC As Gene Therapy For Pancreatic Cancer

**DOI:** 10.1038/s41598-018-37433-6

**Published:** 2019-02-11

**Authors:** Carlotta Spano, Giulia Grisendi, Giulia Golinelli, Filippo Rossignoli, Malvina Prapa, Marco Bestagno, Olivia Candini, Tiziana Petrachi, Alessandra Recchia, Francesca Miselli, Giulia Rovesti, Giulia Orsi, Antonino Maiorana, Paola Manni, Elena Veronesi, Maria Serena Piccinno, Alba Murgia, Massimo Pinelli, Edwin M. Horwitz, Stefano Cascinu, Pierfranco Conte, Massimo Dominici

**Affiliations:** 1 Division of Oncology, Department of Medical and Surgical Sciences for Children & Adults, University-Hospital of Modena and Reggio Emilia, Modena, Italy; 2 Rigenerand srl, Medolla, Modena, Italy; 3 International Centre for Genetic Engineering and Biotechnology, Trieste, Italy; 4 Technopole of Mirandola TPM, Mirandola, Modena, Italy; 5 Department of Life Sciences, University of Modena and Reggio Emilia, Modena, Italy; 6 Department of Diagnostic and Clinical Medicine and of Public Health, Institute of Pathology, University of Modena and Reggio Emilia, Modena, Italy; 7 Aflac Cancer and Blood Disorders Center, Children’s Healthcare of Atlanta and Emory University Department of Pediatrics, Atlanta, GA, USA; 8 Department of Surgery, Oncology and Gastroenerology University of Padova, Istituto Oncologico Veneto IRCCS, Padova, Italy

## Abstract

Pancreatic ductal adenocarcinoma (PDAC) is still one of the most aggressive adult cancers with an unacceptable prognosis. For this reason novel therapies accounting for PDAC peculiarities, such as the relevant stromal reaction, are urgently needed. Here adipose mesenchymal stromal/stem cells (AD-MSC) have been armed to constantly release a soluble trimeric and multimeric variant of the known anti-cancer TNF-related apoptosis-inducing ligand (sTRAIL). This cancer gene therapy strategy was *in vitro* challenged demonstrating that sTRAIL was thermally stable and able to induce apoptosis in the PDAC lines BxPC-3, MIA PaCa-2 and against primary PDAC cells. sTRAIL released by AD-MSC relocated into the tumor stroma was able to significantly counteract tumor growth *in vivo* with a significant reduction in tumor size, in cytokeratin-7+ cells and by an anti-angiogenic effect. In parallel, histology on PDAC specimens form patients (n = 19) was performed to investigate the levels of TRAIL DR4, DR5 and OPG receptors generating promising insights on the possible clinical translation of our approach. These results indicate that adipose MSC can very efficiently vehicle a novel TRAIL variant opening unexplored opportunities for PDAC treatment.

## Introduction

There are more than 150,000 new cases of pancreatic ductal adenocarcinoma (PDAC) diagnosed every year between USA and Europe with an unacceptable 5-years survival rate of 5%^1,2^. Surgery is the first-line treatment, however only 20% of patients are operable and, of those, only 20% survives after 5 years^3,4^. PDAC is relatively resistant to traditional agents, including gemcitabine, 5-fluouracil, taxanes, and platin-derivatives^5^, making the prognosis poor with median survival time reported to be between 5.7 and 11.1 months^6–9^. This still dramatic scenario suggests the need of new approaches capable to take into account PDAC peculiarities. In particular, these tumors generally grow with an abundant hypovascularized stromal reaction both in the primary sites and in the metastases and these high levels of fibrosis are thought to hamper efficacy of the therapeutics^10,11^. Therefore, together with more traditional PDAC targeting agents, strategies able to modify its microenvironment allowing a more performing intra tumor penetration of molecules are demanded. The development of these novel tools aimed at a local deliver of highly active anti-PDAC agents targeting both tumor and his stroma may possibly change the natural history of this still deadly cancer.

Mesenchymal stromal/stem cells (MSC) are adult progenitors that attracted significant interest in cancer research due to their accessibility from different sources together with the possibility of their extensive *in vitro* expansion and gene modification, allowing their pre-clinical and early clinical uses as vehicles for anti-cancer compounds delivery^12–14^. More interestingly, MSC may constitute tumor burden becoming part of the tumor stroma, a property particularly suitable for PDAC targeting^15,16^.

We have previously reported that adipose-derived (AD-) MSC can be used as carrier for anti-cancer agents demonstrating how injected MSC can be localized within tumor microenvironment inducing apoptosis in several cancer types^17,18^. Focusing on AD-MSC delivering a membrane-bound (MB) form of the potent anti-cancer agent tumor necrosis factor (TNF)-related apoptosis inducing ligand (TRAIL), we were able to induce apoptosis in PDAC cell lines *in vitro*^19^. However, that strategy needed a cell-to-cell contact and this may be particularly detrimental *in vivo* where the anti-cancer effect due to MSC may be limited by the tumor bulk requiring strategies to increase TRAIL bioavailability. For this reason, we conceived a novel TRAIL variant capable to be released as a soluble ligand by AD-MSC.

TRAIL is a physiologically-produced protein representing one of the mechanisms by which the immune system reacts against the rise of tumors sparing normal tissues. For this reason the recombinant human (rh) form of TRAIL has been representing a promising antitumor drug^20,21^. Most of PDAC tumor cell lines are sensitive to rhTRAIL^22^, and preclinical evidences suggest that PDAC are sensitive both *in vitro* and *in vivo* to the action of rhTRAIL^23,24^. Combinatory approaches have also indicated relevant synergies between the current chemotherapy agents and rhTRAIL^25^. Different rhTRAIL molecules or TRAIL-receptor agonists have been challenged in pre-clinical and clinical trials, showing a good tolerability but limited therapeutic effects due to several factors, including a very short half-life^26,27^. For all these reasons we sought to combine the MSC affinity for PDAC stroma, their capacity to deliver TRAIL variants and the reported sensitivity of PDAC to rhTRAIL to propose an approach where human MSC are armed by a soluble TRAIL (sTRAIL) and challenged in preclinical models providing evidences of safety and efficacy against a still deathly tumor.

## Results

### Gene modified AD-MSC can secrete a soluble trimeric and multimeric TRAIL variant

**Figure 1:**
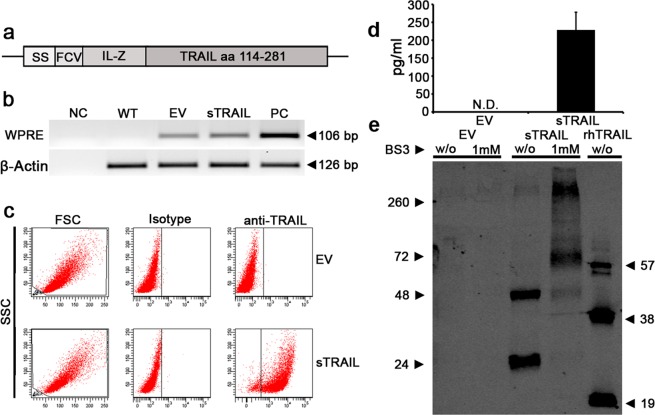
Generation of a secretable trimer-forming variant of human TRAIL. (**a**) The TRAIL expression cassette is here schematically presented. An immunoglobulin-derived secretion signal (SS) is linked to a Furin-cleavage (FCV) domain and to an isoleucine zipper (IL-Z) conjugated with the TRAIL receptor-binding domain (amino acids 114–281). (**b**) Agarose gel showing the amplification (amplicon 106 base pairs, bp) of the lentiviral vector specific sequence WPRE in transduced (empty vector EV and sTRAIL) but not in wild type (WT) AD-MSC. The pCCL-PGK-WPRE vector plasmid was used as positive control (PC) and reaction mix without DNA as negative control (NC). Human β-Actin was introduced as housekeeping control gene in all samples including a known positive control DNA sample from AD-MSC (first lane on the right). (**c**) FACS analyses of gene modified AD-MSC after fixation, permeabilization and staining by an anti-TRAIL antibody to detect intracellular TRAIL. AD-MSC modified with EV are stained as control and, in both cases, cells were stained by isotype control. (**d**) Quantification by ELISA of engineered AD-MSC sTRAIL release (0.2–0.37 femtogram/cell/day). EV AD-MSC sTRAIL production, as expected, was not detectable. (**e**) Western blot analysis for TRAIL expression showing the different size (kDa) of sTRAIL and rhTRAIL. The sTRAIL expression construct cloned in pCCL-PGK-WPRE lentiviral vector has been transiently transfected into 293T cells. Forty-eight hours after transfection culture superntatants were collected, prepared with or without (w/o) adding 1 mM of the BS3 chemical crosslinker. One hundred nanograms of the rhTRAIL were used as positive control, while EV cells were used as negative control. Full-length blots/gels are presented in Supplementary Fig. S9.

The gene encoding for sTRAIL was generated linking different domains (Fig. 1a). Wild type (WT), empty vector (EV) and sTRAIL AD-MSC were first tested by PCR to verify the integration of pro-viral sequences of the lentiviral Woodchuck hepatitis virus post-transcription regulatory element (WPRE) sequence (Fig. 1b). As expected, WT AD-MSC did not generate any amplification, while both EV and sTRAIL AD-MSC confirmed the existence of proviral sequences. Transduction levels in AD-MSC was quantified by FACS revealing that 89.3 ± 5.2% sTRAIL AD-MSC were positive for intracellular TRAIL expression (Fig. 1c). Gene modified AD-MSC cells were then tested for sTRAIL secretion. ELISA tests confirmed that different batches (n = 11) of sTRAIL AD-MSC were capable to release on average 227.8 ± 49.5 pg/ml of TRAIL (Fig. 1d). EV AD-MSC did not spontaneously release sTRAIL.

To further confirm that secreted TRAIL variant was able to trimerize, Western blot analyses were performed to evaluate the molecular weight (MW) of the released TRAIL molecules (Fig. 1e). We focused on supernatants produced by 293T transfected with the sTRAIL-encoding lentiviral vector due to the high level of protein (~36 ng/ml) production and as representative of the structure of the released TRAIL variant. Supernatants were used for Western blot with or without adding the crosslinker BS3^28^. As expected, rhTRAIL showed multiple bands with different intensities: the monomeric, the dimeric and the trimeric forms. Interestingly, without cross-linker, sTRAIL could be detected either as monomer or as dimer with no evidence of a trimeric form while higher multimeric sTRAIL appeared as a weakly visible band at approximately 260 kDa. Conversely, in this Western blot the addition of the cross-linker allowed the identification of a predominant sTRAIL trimer isoform next to a reinforce in the signal of higher MW bands, while monomeric and dimeric forms were present as faint bands. Together these data indicate the generation of a new secretable TRAIL variant prompting its use against PDAC.

### AD-MSC can secrete sTRAIL and maintain their minimal biological properties

sTRAIL AD-MSC and controls were expanded and further characterized to assess whether lentiviral transduction and sTRAIL expression could affect AD-MSC properties.

In all the considered samples, AD-MSC viability by 7AAD staining was 82.2 ± 1.21%. Morphological analyses indicated a classical fibroblastoid shape in all preparations (not shown) and FACS analyses revealed that transduced and WT cells expressed unaltered high levels of CD105, CD73 and CD90, at the same time lacking HLA-DR, CD14 and CD45 (Supplementary Fig. S1). Differentiation assays (bone, fat, cartilage) suggested that AD-MSC gene modification did not impact on the main differentiation lineages, as confirmed by functional *in vitro* studies (Supplementary Fig. S2). Thus, AD-MSC could stably secrete sTRAIL with no apparent interference on their basic properties.

### sTRAIL released by AD-MSC induce apoptosis in PDAC lines and in primary cells by Caspase-8 activation

**Figure 2:**
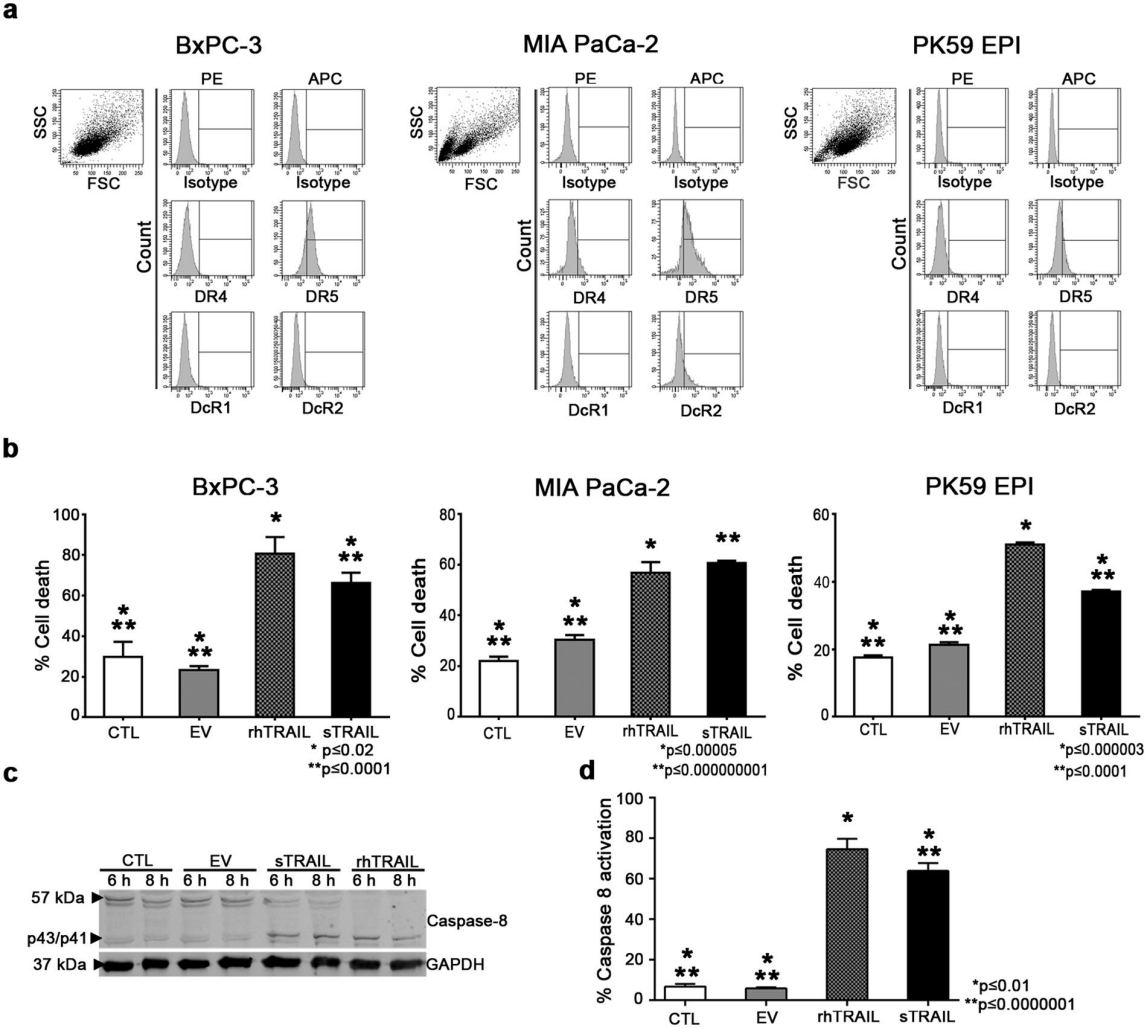
TRAIL receptors and sTRAIL activity on PDAC cell lines. (**a**) Expression of both agonistic (DR4 and DR5) and decoy (DcR1 and DcR2) TRAIL receptors by flow cytometry; DR-4 and DcR1 were phycoerithryn (PE) and DR5 and DcR2 allophycocyanin (APC) stained. Proper isotype controls were used for both fluorochromes. (**b**) Tumor cell death was measured by propidium iodide staining. Recombinant human TRAIL (rhTRAIL; 1 μg/ml) was used as positive control while tumor cell lines with unconditioned control medium (CTL) and empty vector (EV) transduced AD-MSC supernatant were used as negative controls. Reported *p values represent significance of rhTRAIL versus the other groups, while **p refers to soluble TRAIL (sTRAIL) versus controls. (**c**) Western blot analysis on whole cell lysates showing Caspase 8 cleavage in treated (sTRAIL and rhTRAIL) and control (CTL and EV) BxPC-3 cells after both 6 and 8 hours. (**d**) Flow cytometry analysis to detect activated Caspase 8 forms in treated (sTRAIL and rhTRAIL) and control (CTL and EV) BxPC-3 cells after 6 hours of treatment with supernatants and CTL media. Full-length blots/gels are presented in Supplementary Fig. S9.

To evaluate the possible anti-PDAC action by sTRAIL AD-MSC, BxPC-3 and MIA PaCa-2 PDAC cell lines, as well as the primary PDAC cells PK59 EPI, were first tested for TRAIL receptors revealing high levels of DR5, low DcR1/DcR2 expression and a highly variable OPG secretion (Fig. 2a and Supplementary Fig. S3). These relevant levels of DR5 were suggesting a TRAIL sensitivity of selected PDAC lines, as then confirmed in a cytotoxicity assay with increasing doses of rhTRAIL against the selected BxPC-3 cell line (Supplementary Fig. S4). Observing both receptors expression and rhTRAIL sensitivity in PDAC, AD-MSC supernatants containing sTRAIL and controls were collected and used against BxPC-3 (Fig. 2b, left panel). sTRAIL induced a significant cell death versus both unconditioned control medium (CTL) and EV control groups. One μg/ml of rhTRAIL generated the expected apoptosis, even greater than sTRAIL. However we have to underline that, while sTRAIL mean concentration was approximately 8000-fold less (here 121.3 ± 28.8 pg/ml) than the rhTRAIL, the obtained cytotoxicity by sTRAIL was only 0.7-fold less than rhTRAIL. Similarly to BxPC-3, sTRAIL induced 60.6 ± 1.0% of cell death on MIA PaCa-2 cell line (Fig. 2b, middle panel). For the PK59 EPI primary PDAC cells (Fig. 2b, right panel) sTRAIL induced apoptosis in 37.0 ± 0.5% of the cell population versus the 50.9 ± 0.6% of rhTRAIL, the 17.5 ± 0.6% of CTL and the 21.2 ± 0.7% of EV control group.

Once demonstrated the anti-PDAC effect of sTRAIL released by AD-MSC, we wanted to address whether freezing procedures could impact the observed events. sTRAIL containing supernatants were then used either as fresh or as frozen (−80 °C) product (Supplementary Fig. S5). No differences were observed between the two preparations, and conditioned supernatant induced cell death in 66.1 ± 2.5% in BxPC-3 cells if used as fresh and in 62.6 ± 2.5% if used as frozen (p > 0.05). This indicated that sTRAIL activity was not affected by freezing/thawing procedures with values of apoptosis that were significantly higher than controls with conditioned media collected from EV AD-MSC (23.3 ± 0.9% if used as fresh, 24.6 ± 3.2% if used as frozen).

Classically, rhTRAIL induces death via caspase-8^21^, thus we addressed whether sTRAIL released by AD-MSC could similarly induce caspase activation (Fig. 2c) in BxPC-3 cells. SDS-page Western Blot confirmed at molecular level the apoptosis seen by FACS using sTRAIL AD-MSC supernatant and rhTRAIL positive control. In negative controls (CTL and EV), the full-length caspase 8 generated a stronger signal at 57 kDa level while sTRAIL and rhTRAIL showed a more prominent signal at the level of the cleaved forms of the Caspase 8 (p43/p41; between 43 and 41 kDa). An additional read out by FACS was introduced to further confirm the caspase 8 activation after just 6 hours from the sTRAIL-incubation (Fig. 2d). These data collectively indicate that sTRAIL, similarly to rhTRAIL, is able to induce a rapid apoptosis through the activation of caspase 8 pathway.

### sTRAIL anti-cancer impact is due to multimeric sTRAIL molecules generating higher toxicity than rhTRAIL with greater stability at 37 °C

**Figure 3:**
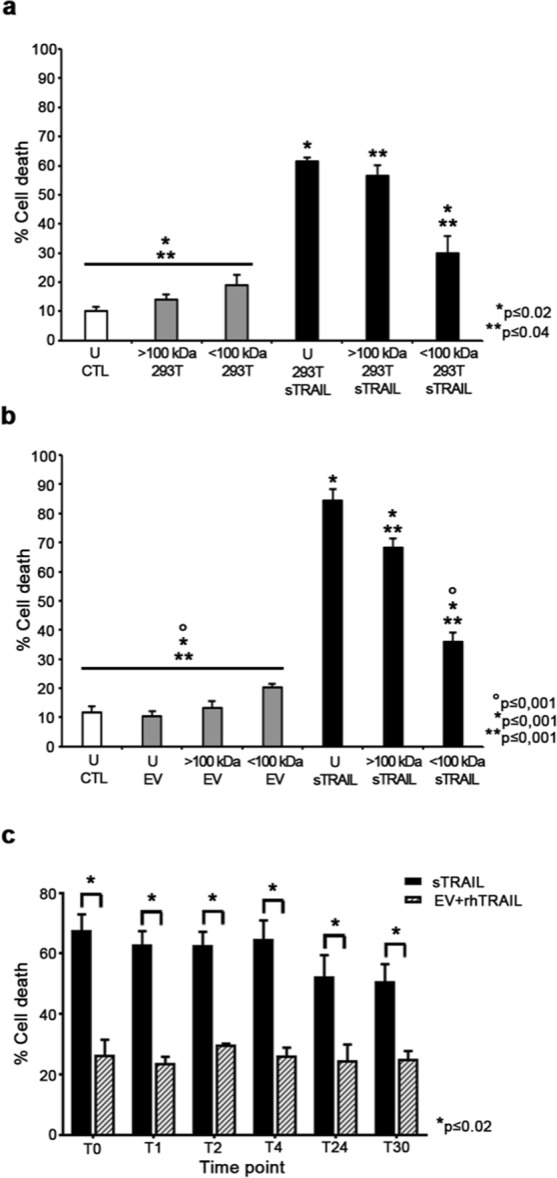
sTRAIL generates multimers with cytotoxic capacity and maintains unaltered its pro-apoptotic activity at 37 °C for 30 hours. (**a**) sTRAIL-mediated cytotoxicity against BxPC-3. Tumor cells were incubated for 24 hours with the upper (>100 kDa) and lower fractions (<100kDa) of sTRAIL-containing supernatant, as well as the unfractionated one (U), collected by sTRAIL 293T. Cytotoxicity was evaluated after 24 hours by FACS using Propidium Iodide (PI) staining. Supernatant deriving from EV 293T was used as control. BxPC-3 cells in normal culture medium (CTL) were evaluated in the same way for comparison. Reported *p and **p values represent significance of the unfractionated sTRAIL supernatant and the >100 kDa sTRAIL supernatants, respectively, versus the other groups. (**b**) Similarly for AD-MSC, we evaluated the cytotoxicity produced by the upper (>100 kDa) and lower fractions ( < 100 kDa) of sTRAIL-containing supernatant, as well as the unfractionated one (U), collected by sTRAIL AD-MSC. Reported *p, **p and °p values represent significance of the unfractionated sTRAIL supernatant, the >100 kDa sTRAIL supernatants and the <100 kDa sTRAIL supernatants, respectively, versus the other groups. (**c**) AD-MSC sTRAIL supernatants were kept at 37 °C for the indicated times points (T = hours) and used for cytotoxicity tests on BxPC-3. PI staining was performed after 24 hours of contact with the supernatants. rhTRAIL diluted at 150 pg/ml into AD-MSC EV supernatant was used for comparison. At each time point sTRAIL activity was superior to the corresponding rhTRAIL control (p ≤ 0.02), no difference was observed at all the time points in terms of anti-BxPC-3 apoptosis induction by sTRAIL.

Since there is increasing evidence that even large multimeric molecular forms of TRAIL receptor agonists and some TRAIL variants could govern bioactivity *in vitro* and *in vivo*^29,30^ we began to explore whether our sTRAIL variant could be associated to higher than trimeric forms, also accounting our just reported data (Fig. 1e). sTRAIL-containing supernatants from both 293T and AD-MSC cultures were fractioned by ultrafiltration to separate molecules with MW > 100 kDa (upper fraction) from molecules with MW < 100 kDa (lower fraction). As visible in Fig. 3a, sTRAIL supernatants, either unfractionated or fractionated, induced a significant cell death compared to control supernatants. Interestingly, the upper fraction was more effective than the lower one with 57 ± 4% and 30 ± 7% cell death respectively (p ≤ 0.04), inducing a mortality comparable to that of unfractionated sTRAIL supernatant (61 ± 1%; p > 0.05). In the same setting, control supernatants had no impact on BxPC-3 viability. When supernatants from AD-MSC cultures were applied (Fig. 3b), unfractionated sTRAIL elicited the expected cytotoxic effect (84 ± 4%; p ≤ 0.001) and, most importantly, cytotoxicity conveyed by the upper fraction appeared again greater than the one by the lower fraction with 68 ± 3% cell death versus 36 ± 3%, respectively (p ≤ 0.001). EV AD-MSC supernatant, both unfractionated or fractionated did not impact on tumor cell survival. These data suggest that cytotoxic activity of our sTRAIL is mainly mediated by high MW multimers, possibly composed by more than three subunits.

In order to evaluate the sTRAIL stability after collection and storage at 37 °C and accounting for literature data on temperature sensitive multimeric and particularly trimeric TRAIL variants^31^, cytotoxicity tests were performed on BxPC-3 using sTRAIL containing supernatants kept at 37 °C for different periods (T0, T1, T2, T4, T24 and T30 hours; Fig. 3c). Since sTRAIL level in the supernatants used for cytotoxicity tests was 150 pg/ml and to verify for the different apoptosis-inducing potential of rhTRAIL versus sTRAIL, the same pg/ml concentration of rhTRAIL was diluted into EV AD-MSC supernatant and used as control. As shown in Fig. 3, at T0, T1, T2 and T4, the sTRAIL-induced BxPC-3 mortality was constantly superior to 60%, slightly decreasing at T24 and T30 with an average value of 59.8 ± 2.8% for all the different points. Anova test did not show significant differences in the cytotoxic potential of sTRAIL, thus demonstrating that prolonged periods of time at 37 °C for sTRAIL containing supernatants did not affect the pro-apoptotic function of sTRAIL. In the EV control media supplemented with 150 pg/ml of rhTRAIL the percentage of cytotoxicity was similar between all the observation times (average 25.6 ± 0.8%) with no difference from the basal mortality obtained with either unconditioned DMEM (CTL) or with EV AD-MSC supernatants previously described in BxPC-3 cytotoxicity assays. At every time point, t-test showed a significant superior killing potency of sTRAIL released by AD-MSC (p ≤ 0.02) in comparison with rhTRAIL given at a similar concentration.

### sTRAIL AD-MSC delivered in mice efficiently control PDAC growth without toxicity

**Figure 4:**
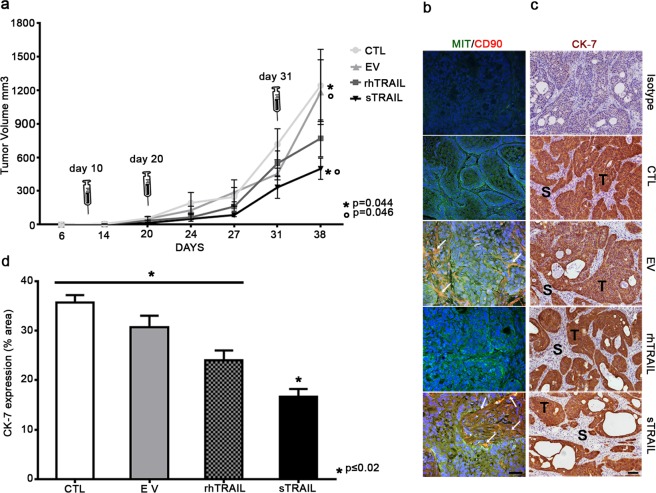
AD-MSC sTRAIL counteract PDAC growth in tumor-bearing NOD/SCID mice. (**a**) Tumor inhibition by three intra-tumor (2 × 10^6^ BxPC-3) administrations (syringe) of 10^6^ AD-MSC sTRAIL. Tumor volume was determined at different time points and at the end of treatment after tumor resection. Values are expressed as mean (±SEM). Difference between rhTRAIL and sTRAIL groups is not significant at the end of the treatment. (**b**) Representative images of anti-CD90/MIT (red DyLight 594/green CF488A dyes) immunofluorescence staining of tumor sections obtained from mice of each group: in CTL, in rhTRAIL and in isotype control group red fluorescence could not be detected. Instead for both EV and for sTRAIL groups, rare human MIT^+^/CD90^+^ cells can be detected (yellow arrows) into the tumor stroma. DAPI was used to stain cell nuclei. Magnification 400x, scale bar 50 μm. (**c**) Representative images of IHC staining for human CK-7 (brown DAB). CK-7^+^ areas represent tumor islets (T) as this marker is typically expressed by PDAC cells. Tumor stroma (S) with fibroblastoid murine cells was constantly negative for CK-7. Magnification 100x, scale bar 100 μm. (**d**) Histogram representing the quantification of CK7^+^ areas of panel C within the different groups performed by Image-J.

To verify whether the tumoricidal activity of sTRAIL AD-MSC could be reproduced *in vivo*, a xenotransplant *sub cutis* model was generated by injection of 2 × 10^6^ BxPC-3 cells. Tumor burdens became appreciable 6 days from the inoculum and 3 doses of EV AD-MSC or sTRAIL AD-MSC (1 × 10^6^ cells/each) were peritumorally injected at days 10, 20 and 31 (Fig. 4a). For comparison rhTRAIL was intravenously injected with the same dose and schedule according to previously reported data (5 mg/Kg)^18^. Mice treated with sTRAIL AD-MSC had tumors whose average size was considerably lower than CTL and EV controls, indicating that sTRAIL *in vivo* produced by AD-MSC was able to significantly inhibit tumor growth. Despite tumor growth was less prominent in the rhTRAIL group when compared to control groups (CTL and EV), no statistically significant differences could be detected at the end of treatment.

To additionally assess the safety of this TRAIL-based anticancer approach, mice health was monitored considering animal weight, food intake and behaviour and no differences between groups were observed. Animal weight was weekly monitored and no significant difference could be observed during treatments (Supplementary Fig. S6a). Based on the reported hepatic toxicity of TRAIL^32^ at the moment of sacrifice mice livers were collected and paraffin embedded for subsequent H&E staining. No apparent effects could be observed in all groups thus indicating the safety of the approach in this pre-clinical model (Supplementary Fig. S6b).

### sTRAIL AD-MSC survive after peritumoral delivery decreasing CK-7 levels and angiogenesis *in vivo*

The engraftment of gene-modified AD-MSC into tumor masses has been evaluated by immunofluorescence through the staining of human CD90 (Fig. 4b, in red) as differential marker between mice-derived stromal elements and human AD-MSC, and MIT (Fig. 4b, in green) as marker for both tumor and MSC cells of human origin. The presence of rare mesenchymal elements in tumor stroma demonstrates the capacity of AD-MSC to survive and engraft for at least 7 days after the last implant (Fig. 4b, in yellow, as a result from the merging between CD90 and MIT in EV and sTRAIL groups). AD-MSC were exclusively found in tumor stroma surrounding cancer tissue with no evidence of intra-PDAC localization.

In addition to the measurement of BxPC-3 tumor size and engraftment, *in vivo* experiments allowed focused histological analyses of the PDAC xenotransplant specimens. Therefore, for each group, tumor composition was evaluated looking at CK-7 positive area (Fig. 4c), accounting that PDAC classically retains a relevant desmoplastic/stromal reaction that may generate an underestimation of the sTRAIL impact on PDAC xenotransplants size^10^. As expected, CK-7 was specifically staining PDAC cells (in brown) and not stroma (in blue) that predominantly appeared of murine origin being also MIT- (Fig. 4b). Histology confirmed the presence of an abundant stromal compartment in all groups (in blue) and, interestingly, sTRAIL AD-MSC treated group gave a greater evidence of multiple large empty spaces in the tumour parenchyma (Fig. 4c, lower panel). Considering the CK7 positive areas only, mice treated by rhTRAIL and sTRAIL revealed less prominent PDAC islets. Confirming these qualitative data and the tumor volume assessments, the Image J scoring of CK-7+ areas (Fig. 4d) indicated that sTRAIL treated animals have a significantly lower PDAC cell number versus all the other groups.

Since TRAIL has been previously described to inhibit angiogenesis^33,34^ and based on our previous findings^18^, we then investigated PDAC xenograft vascularization. Tumor sections were stained by anti-CD31 antibody to compare the presence of vessels in the different conditions (Supplementary Fig. S7a). sTRAIL was able to significantly decrease the number of vessels compared to control groups, similarly to rhTRAIL group. To confirm the anti-angiogenic effect observed in our *in vivo* model, an *in vitro* cytotoxicity assay has been performed on primary HUVEC cells (Supplementary Fig. S7b). After 24 hours of incubation, sTRAIL AD-MSC supernatants were able to significantly induce HUVEC cell death compared to both rhTRAIL and control groups. Collectively, these findings confirmed *in vivo* the role of sTRAIL released by AD-MSC as a potent anti-PDAC agent capable to act directly on tumor cells and as influencing player within the PDAC microenvironment.

### TRAIL receptors DR4, DR5 and OPG are expressed in human-derived PDAC

**Figure 5:**
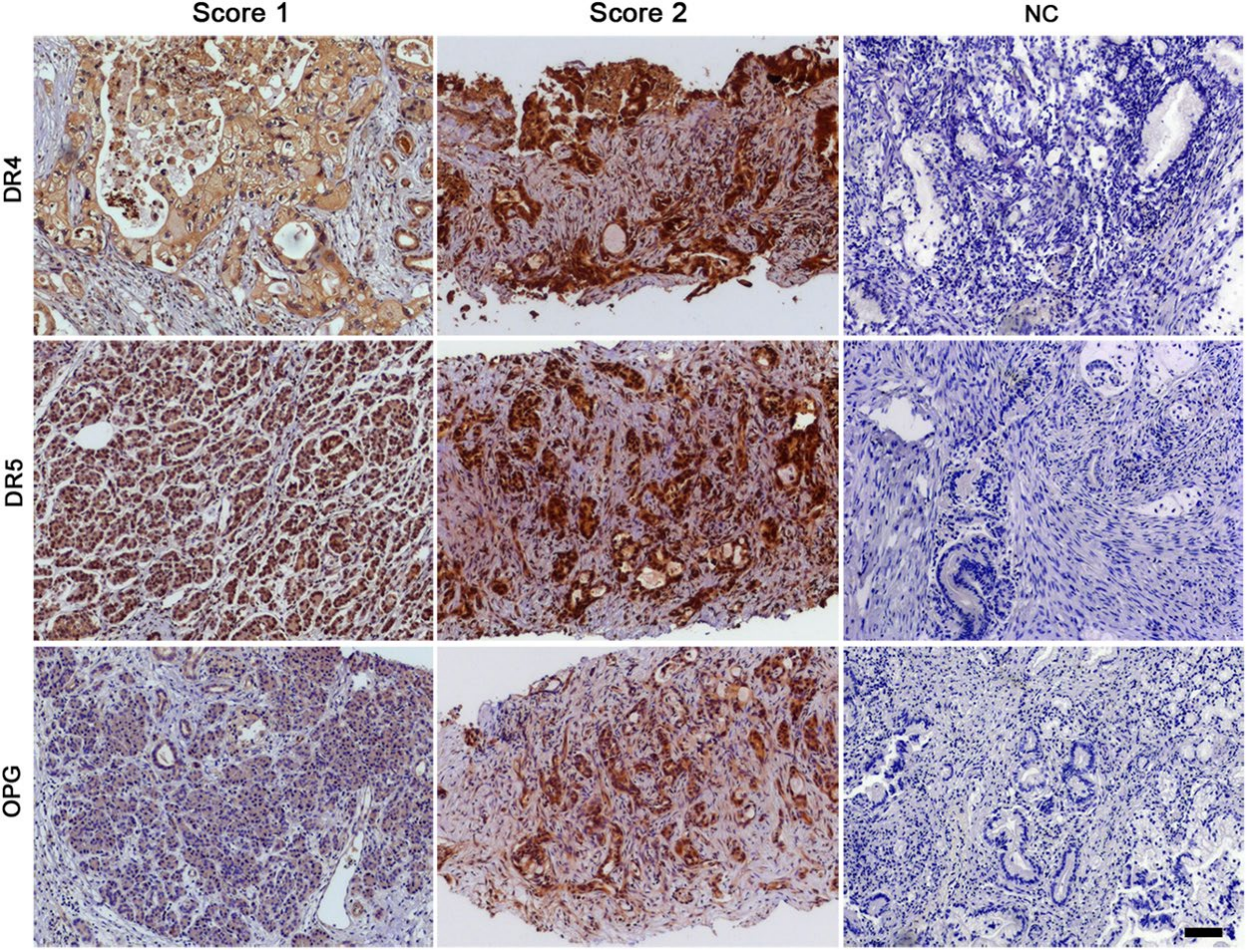
Tissue samples from human PDAC express TRAIL receptors. Representative images of the staining with antibodies anti- DR4, DR5 and OPG. All TRAIL receptors were expressed *in vivo* in PDAC specimens from patients. Different intensities of expression (classified as score 1 or 2) have been observed for each receptor. Samples stained by omitting primary antibody were used as negative controls (NC). Magnification 100x, scale bar 100 μm.

Taking into account these pre-clinical data and considering the relevance of addressing TRAIL receptors expression (DR4, DR5 and OPG) in human PDAC specimens, a group of 19 patients affected by PDAC (n = 10 from Computed Tomography/ecoguided biopsies and n = 9 from pancreatectomy; histological grading: n = 6 G3 and n = 13 G2) was considered (Fig. 5). DR4 and DR5 were positive in 19/19 patients (100%). Since OPG is a secretable protein physiologically expressed by vessel structures^35^, we introduced a 10% cut-off to discriminate between positive and negative specimens. By applying this strategy 11/19 (58%) patients were positive for OPG expression and 8/19 (42%) were negative. Although DR4, DR5 and OPG were expressed in the majority of the cases, different intensities of staining were observed. Thus, we have developed a semi-quantitative scoring system^36^, to distinguish negative samples (score 0) from those weakly-moderately (score 1+) or strongly (score 2+) positive. Based on this approach different results have been obtained for each receptor. For DR4, 6/19 (32%) patients received score 1+ while 13/19 (68%) received score 2+. For DR5 a similar distribution has been observed with 7/19 (37%) score 1+ and 12/19 (63%) score 2+. Applying the scoring system to OPG staining, 8/19 (42%) samples were classified in score 0, 9/19 (47%) belonged to score 1+ and 2/19 (11%) to score 2+. In Fig. 5 is reported a representative panel of weakly-moderately (score 1) and intensely (score 2) positive samples and negative controls (NC) for each receptor.

## Discussion

PDAC still remains one of the deadliest form of solid tumors and, despite surgery, radiotherapy and chemotherapy combinations, patient survival has been minimally improved^37,38^ indicating the need for novel and more effective therapeutic strategies. We here originally show the possibility to counteract human PDAC growth using gene modified human AD-MSC expressing a soluble TRAIL variant. MSC are known for their ability to infiltrate tumor stroma^15,16^, a component particularly relevant in PDAC^39,40^. TRAIL is a TNF superfamily member able to exert a pro-apoptotic action towards TRAIL-sensitive cancer cells, while sparing normal cells^32^. We armed AD-MSC with a soluble form of TRAIL to generate a constant release of TRAIL into tumor microenvironment and to overcome the known low TRAIL bioavailability^41^.

The gene encoding for the soluble TRAIL was synthetized by linking different domains and cell modification was confirmed by the capacity to release soluble TRAIL. In the past, others generated soluble TRAIL variants produced by *E. coli*, *P.* *pastoris*, the 293T cell line, by intra-tumor delivered adenoviral vector, by delivering of sTRAIL gene via nanoparticles targeting tumor stroma, by engineered (adeno- or lenti-viral vectors) human, rat and murine bone marrow MSC and by transfected mesenchymal cells derived from pancreatic tissue^31,32,42–51^. All these efforts aimed to increase TRAIL bioavailability to ultimately generate apoptosis. Here, we were able to generate a sTRAIL variant showing all forms of the released molecule, from monomers to multimers that demonstrated a significant cytotoxicity in comparison to rhTRAIL at the same dose. It is known that biologically active trimerization is necessary to generate a more efficient apoptosis^52^ and it has been reported that even higher multimeric TRAIL forms are associated with an enhanced cytotoxic activity^29,30^. We sought to generate AD-MSC releasing a sTRAIL capable of a robust cytotoxicity by multimerization. While the exact structure of these forms shall require a dedicated study, we originally report that a predominant anti-PDAC action is due to TRAIL structures having a molecular weight higher than 100 KDa, therefore theoretically more than TRAIL trimers. An additional explanation for the better sTRAIL action may be linked to different polymerization domain of our sTRAIL versus rhTRAIL. Based on wild type or rhTRAIL mechanism of action, Zn^2+^ concentration seems to be critical for the biological activity and in particular for trimerization^53^. In fact, without the Zn^2+^, TRAIL slowly converts into inactive monomeric or dimeric forms^54^. In our case, the use of an isoleucine zipper in the sTRAIL overcomes the need of Zn^2+^^55^ for multimerization versus rhTRAIL accounting for the superior cytotoxic activity.

We previously gave evidence that a MB form of TRAIL expressed by gene modified AD-MSC can induce apoptosis in tumor cell lines, including PDAC, by a cell-to-cell contact^17,19^. Starting from that experience we developed a sTRAIL form able to exert its cytotoxic effect without the need of AD-MSC contact with cancer cells. This seemed particularly valid for PDAC that is characterized by a dense fibrotic microenvironment that makes hardly accessible tumor cells for AD-MSC contacts. 

Due to the presence of TRAIL receptors in AD-MSC^17^, we also verified that the release of multimeric sTRAIL did not impact their biological properties by an autocrine loop. Our data suggest that gene modification is not compromising AD-MSC features including proliferation (not shown), immunophenotype or differentiation, confirming the TRAIL resistance observed in other mesenchymal progenitors^56^ and further demonstrating the possibility of AD-MSC to be a clinically relevant vehicle for TRAIL^17,43,57^.

While there was no impact on AD-MSC themselves, we were able to induce apoptosis by caspase-8 activation in PDAC cell lines expressing death receptors and reported to be sensitive to TRAIL-induced apoptosis^58^. Moreover, for the first time human primary PDAC cells were challenged by sTRAIL supernatants produced by human AD-MSC. The results are encouraging also considering the abundant amount of decoy receptor OPG produced by these cells and taking into account that primary PDAC may be relatively resistant to rhTRAIL^59,60^.

Interestingly, in most cases the supernatants used for cytotoxicity assays were subjected to a freezing (−80 °C) cycle, without any significant loss of sTRAIL activity, indicating the stability of this TRAIL variant at low temperatures. In addition, when we considered the concentration of rhTRAIL with the sTRAIL, we observed that the cytotoxic potential of the latter was significantly superior presumably due to a greater stability at 37 °C of the sTRAIL versus the rhTRAIL, as reported^31,52^. This hypothesis has been also confirmed by the evidence that an induction of apoptosis against PDAC cells could be persistently obtained using supernatants harvested from modified AD-MSC and kept at 37 °C for up to 30 hours before cytotoxicity assays.

Starting from those encouraging *in vitro* data we explored the anti-tumor potential of sTRAIL AD-MSC *in vivo* showing that peritumoral injection of sTRAIL AD-MSC were able to control tumor growth and rare AD-MSC were found in tumor stroma for at least 7 days after injection, similarly to what has been described using non-engineered AD-MSC to treat PDAC^61^.

Moreover, considering the xenotransplanted tumors architecture and accounting for the CK-7 staining, it was possible to observe how PDAC islets were significantly reduced by TRAIL with a histological pattern characterized by empty spaces and a more conspicuous stromal component. A further observation on tumor specimens was related to the impact of TRAIL in angiogenesis since it is known that TRAIL may exerts an impact onto tumor vascularization^18,33^. The number of vessels was significantly reduced both in mice treated with sTRAIL and with rhTRAIL. Of note, rhTRAIL showed *in vivo* a similar effect on vessel number compared to sTRAIL. This may be apparently in contrast with the reduction of CK7+ PDAC seen in histology by sTRAIL AD-MSC versus rhTRAIL. However, the overall reduction of PDAC cells is likely dependent also on sTRAIL direct cytotoxic effect and thus on sTRAIL presence inside the tumor with a persistence of the proapoptotic stimulus. To this end, we can hypothesize that intravenously administered rhTRAIL is able to efficiently reach tumor vessels but its diffusion inside the tumor mass may be hindered by the dense stromal component and, most importantly, by rhTRAIL very short half-life (3–5 minutes in rodents)^62^ with a limitation due to its bioavailability. On contrary, MSC are administered intra-tumor and can still be found at the end of the treatment (as visible in Fig. 4b) thus providing a continuous supply of sTRAIL inside the tumor burden which, even at lower effective concentrations than rhTRAIL, may contribute to its superior performance. While the anti-angiogenetic impact of sTRAIL shall require further studies, the *in vivo* findings were here further confirmed *in vitro* against human endothelial cells (HUVEC), where sTRAIL confirmed its anti-angiogenetic potential, even higher compared to rhTRAIL. Therefore, sTRAIL seems to have double effect in this model generating PDAC cell death and in reducing angiogenesis.

Since TRAIL variants have been previously associated with liver toxicity^63^, the animal model gave also the opportunity to demonstrate that multiple doses of AD-MSC sTRAIL were related with neither hepatotoxicity nor other clinical relevant side effects, as previously reported by our group with MB TRAIL and by others with sTRAIL^17,18,64^.

With the limitation of a *sub cutis* xenotransplant, our animal model gave important insights on the effect of sTRAIL delivered by AD-MSC on PDAC without exploring cell migration and homing. In fact, subcutaneous PDAC xenografts, are relatively poorly vascularized and usually comprise an abundant stromal reaction that may hamper an intratumor migration after intravenous injection. Addressing homing would require additional studies within an orthotopic tumor model able to better mimic the clinical scenario possibly to be targeted by either intratumor or intravenous injections also accounting the recent evidences of intravenously infused cells trapped and destroyed into the lungs^65,66^, with a possible reduction on their therapeutic profile.

Having demonstrated by *in vitro* and *in vivo* models the potential of sTRAIL released by AD-MSC we began to consider the distribution of the two functional TRAIL receptors in a cohort of 19 PDAC patients, as a prerequisite for a clinical translation. TRAIL acts as a homotrimer through the interaction with its two functional receptors DR4 and DR5 that are essential for apoptosis signaling^67–69^. Interestingly, DR4 and DR5 were highly expressed in all our samples similarly to what described by Stadel *et al*. in a group of 24 patients. They previously demonstrated the very relevant prevalence of both DR4 (91%) and DR5 (75%) expression in the considered cohort, suggesting the possible clinical responsiveness to TRAIL in PDAC^70^. Others recently described how low levels of TRAIL receptors in PDAC seems to be associated with a worse prognosis, suggesting that the lack of TRAIL receptors could represent an escape mechanism for a TRAIL induced apoptosis^36^.

We additionally considered OPG as pivotal TRAIL decoy receptors that binds TRAIL inhibiting its apoptosis inducing potential^71,72^. More than a half of the examined cases gave evidence of OPG expression. Similarly, Shi *et al*. confirmed OPG overexpression in PDAC samples indicating that increased expression of OPG in PDAC tissues correlated with a poor overall survival^73^. These data suggest the relevance of TRAIL/OPG axis in PDAC progression indicating how the introduction of therapeutic tools capable to interfere in this pathway may generate a clinical benefit.

In conclusion, while further investigations shall be requested on cell dose and schedule together with the possibility to introduce combinatory approaches with more traditional chemotherapy agents, our data demonstrate that AD-MSC can be modified to efficiently release sTRAIL inducing PDAC death in different pre-clinical models persisting into PDAC xenotransplants sufficiently to impact against tumor growth. These findings along with the histology data from patients indicate the possibility to induce death by a sTRAIL secreted by *ex vivo* modified adult progenitors targeting a cancer type with still unacceptable survival rates.

## Materials and Methods

### Cell cultures

The human kidney embrionic 293T cells, were cultured in DMEM (GIBCO, Thermoscientific, Waltham, MA, USA) with 10% heat inactivated defined FBS (HyClone Laboratoires, Inc, Logan, Utah, USA), 2 mM L-Glutamine (BioWhittaker, Lonza, Verviers, Belgium) and 1% penicillin/streptomycin (pen/strep, Carlo Erba Reagents Srl, Cornaredo, Italy). Human PDAC cell lines BxPC-3 (Interlab Cell Line Collection, ICLC, Genova, Italy) were maintained in RPMI (GIBCO) with 10% heat inactivated FBS (Carlo Erba Reagents Srl) and MIA PaCa-2 (ATCC, LGC Standards S.r.l., Milan, Italy) were cultured in DMEM 10% FBS, 2,5% horse serum (Euroclone SpA, Milan, Italy), 1% pen/strep and 2 mM L-Glutamine. Primary human PDAC line defined as PK59 EPI (a kind gift from Valeria Sordi, S. Raffaele Scientific Institute, Milan, Italy) was maintained in a medium composed by DMEM/RPMI (1:1), 10% FBS, 1% L-Glutamine and pen/strep, 1% NEEA, 1 mM NaPiruvate, 2.9 mg/L Insulin, 1 μM Ossalacetic Acid (all by Sigma Aldrich, St. Louis, MO, USA). PK59 EPI were isolated from a patient diagnosed with PDAC. Cells were characterized for epithelial cell adhesion molecule (EpCAM) and Cytokeratin 7 (CK-7) expression (Supplementary Fig. S8). Human Umbilical Vein Endothelial Cells (HUVEC) (Cascade Biologics, Portland, OR) were maintained in culture using M-200PRF medium supplemented with LSGS Kit (both from GIBCO) and 1% antibiotic (pen/strep). In the full respect of national guidelines/regulations and after approval by the Modena Ethical Committee on human studies (http://www.aou.mo.it/ComitatoEticoProvinciale), human AD-MSC were isolated (after obtainment of the informed consent) from healthy donors and expanded in maintenance medium consisting of α-MEM without nucleosides (GIBCO) supplemented with 2.5% of buffy coat-derived pooled platelet lysates (PL; from the Policlinic of Modena Blood Bank, Modena, Italy), 1% L-Glutamine, 1 UI/mL heparin (Sigma Aldrich), and 10 μg/mL ciprofloxacin (Fresenius Kabi Italia S.r.l., Verona, Italy), as described^74^. Tumor cell lines authentication was performed by DNA profiling using 8 different and highly polymorphic short tandem repeat (STR) loci (DSMZ-Authentication Service, Braunschweig, Germany).

### Viral vectors and AD-MSC transductions

An immunoglobulin secretion sequence (SS)^75^, the human stromelysin-3 furin-specific cleavage site (FCV)^76^, the yeast GCN4 isoleucine zipper trimer-forming domain (IL-Z)^55^ were combined with the TRAIL receptor binding domain sequence (Apo2L; amino acid 114–281) using a splicing by overlap extension (SOE) by PCR^75^. Properly sequenced sTRAIL gene has been then cloned (XmaI/SalI) into the lentiviral vector pCCL-PGK-WPRE to gene modify AD-MSC. EV transduced AD-MSC were used as control. The 293T cells were then transfected using JetPEI DNA transfection reagent (Polyplus transfection, Illkirch, France) following manufacturer’s instructions using a combination of lentiviral vector and helper plasmids: pRSV.REV, pMD2.VSVG (envelope), pMDLg/pRRE (gag/pol elements). One ml of lentiviral supernatant produced by transfected 293T supplemented with 6 μg/ml polybrene (Sigma Aldrich) was used to transduce 45000 AD-MSC at early passages (p2-p4). Infections were repeated twice followed by cell expansion and characterization. Viral supernatants were used either as fresh or as −80 °C frozen product.

The viral integration into AD-MSC has been determined by genomic DNA extraction using QIAamp DNA Mini Kit (Qiagen GmbH, Venlo, Limburg, Netherlands) performing a PCR on 100 ng of template DNA using WPRE forward 5′-CGCTGCTTTAATGCCTTTGTAT-3′ and WPRE reverse 5′-GGGCCACAACTCCTCATAAA-3′ primers (95 °C 1′, 95 °C 15″–52 °C 15″–72 °C 30″ for 30cycles, 72 °C 10′), able to detect the lentiviral WPRE by generating an amplicon of 106 base pairs both in the EV and in the sTRAIL AD-MSC. Wild type AD-MSC DNA were used as a negative control. The pCCL-PGK-WPRE EV plasmid has been used as positive control. Human β-Actin housekeeping gene has been amplified in parallel using forward 5′-GGCATGGGTCAGAAGGATTC-3′ and reverse 5′-GTGCCAGATTTTGTCCATGTC-3′ primers, generating a 126 bp amplicon. In both cases amplified products were resolved with ethidium bromide (Euroclone) 1,5% agarose gel (Eurobio, Les Ulis, France) and visualized by ChemiDoc XRS+ (Biorad Laboratoires, Inc, California, USA).

### Protein assays

To characterize sTRAIL molecular weight, Western blot analyses were performed on sTRAIL 293T supernatants that contained significant levels of sTRAIL measured by ELISA (Quantikine Human TRAIL/TNFSF10 kit; R&D Systems, Inc, Minneapolis, MN, USA). 293T supernatants were obtained replacing transfection medium after 24 hours from the transfection with pCCL-PGK-WPRE-sTRAIL vector. The collection medium was composed by DMEM without phenol red (GIBCO) with 1% L-Glutamine and 1% pen/strep. Forty-eight hours after transfection, 293T supernatants were collected and analyzed. EV 293T supernatants and 100 ng of rhTRAIL/Apo2 Ligand (rhTRAIL) (Peprotech Inc., Rocky Hill, NJ, USA) were used as negative and positive controls, respectively.

Thirty μl of supernatants from EV and sTRAIL 293T were loaded on 4–20% MiniProtean-TGX Stain Free (BioRad Laboratoires) for SDS-PAGE with or without 1 mM BS3 (bis[sulfosuccinimidyl]suberate; Thermoscientific) chemical cross-linker^28^. TRAIL was then detected by a rabbit anti-human TRAIL/Apo2 (Peprotech Inc.) followed by a secondary goat anti-rabbit IRDye 800CW (LI-COR, Lincoln, NE, USA) antibody. Chameleon Duo Pre-Stained protein ladder (2 μl, LI-COR) has been used as molecular weight marker. Signals were then captured by the Odyssey Infrared Imaging System (LI-COR). To evaluate the levels of sTRAIL in both EV and sTRAIL AD-MSC, cells were seeded and PL-based culture medium was replaced with DMEM with 10% heat inactivated defined FBS, 1% L-Glutamine and 1% pen/strep. After 48 hours, supernatants were collected from confluent cultures and analyzed by ELISA (Quantikine Human TRAIL/TNFSF10 kit) according to instructions.

### TRAIL and TRAIL receptors by FACS

TRAIL expression in transduced sTRAIL AD-MSC and EV controls was assessed by FACS. Briefly, after fixation and permeabilization cells were stained (BDCytofix/Cytoperm, Bd Biosciences, San Diego CA, USA) with APC-conjugated anti-human TRAIL anti-body (APC anti-human CD253 - TRAIL, Biolegend, San Diego, CA, USA) and isotype control (APC Mouse IgG1, k isotype CTRL, Biolegend). TRAIL receptors expression on tumor cell lines and primary tumor cells was tested by FACS. Samples were harvested and stained with PE-conjugated anti-TRAIL-R1/DR4 and anti-TRAIL-R3/DcR1, APC-conjugated anti-TRAILR2/DR5 (Biolegend) and anti-TRAIL-R4/DcR2 (R&D Systems) with appropriate PE (BD Pharmigen, San Diego, CA, USA) and APC (Miltenyi Biotec Inc., Auburn, CA, USA) isotype controls. Analyses were performed with FACS Aria-III (Becton Dickinson, Franklin Lakes, NJ, USA). Collected data were elaborated by FACS Diva software (Becton Dickinson).

### Apoptosis and caspase-8 activation assays

Target PDAC cells (BxPC-3, MIA PaCa-2 and the primary tumor cells PK59 EPI) were seeded (2 × 10^4^/well in 12 well plates) and after 18 h, either sTRAIL AD-MSC or EV AD-MSC conditioned supernatants (1 ml/well) were added to PDAC cultures. In particular, filtered (0.22 μm, Corning Incorporated, Corning, NY, USA) supernatants were collected from confluent cultures of transduced AD-MSC kept for 48 hours in DMEM 10% FBS. Fresh or −80 °C frozen products were tested against the BxPC-3 for comparative analyses, while only frozen supernatants have been used againts MIA-PaCa-2 and PK59 EPI lines. Activity of sTRAIL produced by AD-MSC on PDAC cells was evaluated after 24 hours by FACS using propidium iodide (PI; 50 μg/ml; Sigma Aldrich) supravital staining. rhTRAIL (1 μg/ml; Peprotech Inc.) was used as positive control. Fresh medium and EV supernatants were used as negative controls. Experiments were performed at least 2 times in duplicate.

To assess the stability in the anti-PDAC cytotoxic action of sTRAIL at 37 °C, conditioned supernatants were separated and filtered (0.22 μm) from sTRAIL producing AD-MSC after 48 hours of culture. Those supernatants were kept at 37 °C and then introduced after 0, 1, 2, 4, 24 and 30 hours for cytotoxicity assays (performed after 24 hours of culture by PI staining against BxPC-3). EV supernatants supplemented by rhTRAIL (150 pg/ml) were used for comparisons.

The Caspase-8 activation was observed by Western blot on BxPC-3 cell lysates, as previously described^18^. Briefly, BxPC-3 were seeded at 4000/cm^2^ in 6-well plates 16 hours before the treatment with sTRAIL. PDAC cultures were treated with sTRAIL supernatant or with rhTRAIL (1 μg/ml) as a positive control. Negative controls were represented by unconditioned DMEM (CTL) with 10% heath inactivated defined FBS and by the EV frozen supernatant. Cells were harvested and lysed after 6 or 8 hours of treatment with conditioned media and controls. Protein lysates were quantified using Biorad Protein Assay (BioRad Laboratoires,) for the measurement at 595 nm by spectrophotometer (GeneQuant pro, Amersham Biosciences, Freiburg, Germany). Thirtyfive μg of total protein obtained from cell lysates have been loaded on 12% MiniProtean-TGX (BioRad Laboratoires, Inc, California, USA) for SDS-PAGE. After the transfer two blots have been performed: the first for the detection of Caspase-8 using Caspase 8 (1C12) Mouse mAb (Cell Signaling Technologies, Beverly, MA, USA) as primary antibody and goat anti-mouse IRDye 800CW (LI-COR) as secondary antibody. The second blot was then performed to detect GAPDH using GAPDH (14C10) Rabbit mAb as primary antibody and Goat anti-Rabbit IRDye 800CW (LI-COR) as secondary antibody. Evaluation of Caspase 8 activation was also done by FACS in BxPC-3 cancer cell line treated with sTRAIL. After 6 hours of culture with medium/supernatants, FACS analysis of BxPC-3 cells measured caspase 8 cleavage in treated (sTRAIL and rhTRAIL) and controls (CTL and AD-MSC EV) cells after 6 hours of culture with medium/supernatants. The staining has been performed using Vibrant FAM Caspase-8 Assay Kit (Molecular Probes, Life Technologies, Carlsbad, CA, USA) following the manufacturer’s instructions.

### Supernatant fractionation and cytotoxicity assay

Supernatants from EV and sTRAIL-transfected 293T were collected as previously described and filtered with 0.22 μm syringe filter (Corning Incorporated). sTRAIL was then quantified by ELISA and diluted by 100 folds to obtain a concentration comparable to supernatants from sTRAIL AD-MSC cultures. Supernatants were then concentrated by membrane ultrafiltration using the Vivaspin® 100 kDa MWCO sample concentrators (Sigma Aldrich). The process was performed in a single 50 ml conical tube with an upper compartment containing the sample, separated from a lower compartment by a semipermeable membrane with a MW cut-off of 100 kDa. Centrifugation was performed at 3800xg for 25 minutes to force supernatant through the membrane. Molecules with a MW higher than 100 kDa (MW > 100 kDa) are withheld in the upper compartment while smaller molecules (MW < 100 kDa) are pulled in the lower one. Supernatants from EV and sTRAIL AD-MSC were collected from confluent cultures, kept 48 hours in α-MEM 2,5% PL and concentrated with the same protocol. The two fractions were used in a cytotoxicity assay against PDAC cell lines. Briefly, BxPC-3 were seeded at 2 × 10^4^ cells/well in 12 well plates and, after 18 hours, culture medium was substituted with either the upper and the lower fractions of the supernatant collected from 293T or AD-MSC (1 ml/well). Cell viability was evaluated after 24 hours by FACS using PI staining. Fresh medium and EV supernatants were used as negative controls.

### *In vivo* studies

Male and female NOD.CB17-Prkdc^scid^/J mice (Charles River, Lecco, Italy) were kept in accordance with guidelines and under approved protocols by the Local Ethical Committee on Animal Experimentation and by the Italian Ministry of Health. Four groups of mice (n = 7/each) were established as follows: (1) sub-cutaneously flank injected (s.c.f.i) with 2 × 10^6^ BxPC-3 (tumor only control group, CTL) in 200 μl Phosphate Buffered Saline (PBS, Biochrom, GmbH, Berlin, Germany); (2) s.c.f.i with 2 × 10^6^ BxPC-3 and, as soon as an appreciable tumor burden appeared (6 days, 0.4–0.5 mm^3^), treated with multiple (n = 3) peri-tumoral injections of 10^6^ AD-MSC EV (EV group) in 200 μl PBS every 10 days; (3) tumor injected as in (2) but treated with multiple (n = 3) peri-tumoral injections of 10^6^ AD-MSC sTRAIL (sTRAIL group) in 200 μl PBS, (4) tumor injected as in (2) but treated with multiple (n = 3) tail intra-venous (i.v.) injections of rhTRAIL/Apo2 Ligand (Peprotech Inc.): 5 mg/Kg, (125 μg/mouse) in 200 μl PBS (rhTRAIL group). Parameters such as survival and weight were monitored. In all groups, weights were weekly recorded, tumor sizes were measured with a calliper and volumes were calculated as reported^37^: volume = length × width^2^/2. After 38 days, animals were sacrificed and tissues were harvested for histology.

### Animal and human histology

Formalin-fixed, paraffin-embedded tumor sections were evaluated by hematoxylin-and-eosin staining (Sigma-Aldrich). For immunohistochemistry analysis, sections were retrieved in citrate buffer (pH 6) for 15 minutes and incubated overnight at 4 °C with the primary antibody rabbit monoclonal anti-cytokeratin 7 (CK-7) SP52 (Ventana, Tucson, Arizona, USA) to confirm PDAC origin. Slides were then incubated with a biotinylated goat anti-rabbit IgG (H + L) (1:200; Vector Laboratories, Burlingame, CA) for 1 hour at room temperature. Negative controls were run simultaneously omitting primary antibody while incubating with buffer. Staining was performed and visualized by 3.3 O-diaminobenzidine (DAB) (in brown, Vector Laboratories). All slides were counterstained with Harris hematoxylin (Bio Optica, Milan, Italy). CK-7 expression was analyzed with ImageJ software (NIH, Bethesda, MD) considering 10 fields for every animal of each group.

For immunofluorescence, tumor sections were deparaffined and retrieved in citrate buffer (pH 6) for 15 minutes. Tissues were permeabilized with Tryton x100 (Sigma Aldrich) for 3 minutes on ice and incubated with primary monoclonal anti-CD90 antibody (1:100; ab133350, Abcam) and then with Goat anti-Rabbit IgG (H + L) secondary antibody, DyLight 594 conjugate (1:700, Thermo Scientific) for 1 hour at room temperature each.

Double staining was performed on slides that were first incubated with anti-human mitochondria antibody (MIT) (1:100; MAB1273, Millipore Corporation, Billerica, MA, USA) and then with CF488A Goat Anti-Mouse IgG (H + L) antibody (1:1000, Biotium, Corporate Place Hayward, CA, US) for 1 hour at room temperature each. Subsequently, slides were mounted in Fluoroshield with DAPI (Sigma Aldrich). Micrographs were taken on Axio Imager M.2 Fluorescent Microscope (Zeiss).

TRAIL receptors expression in human histologically confirmed PDAC specimens (n = 19) was tested in collaboration with the Division of Pathology of the University of Modena and Reggio Emilia after Modena Ethical Committee approval. Formalin-fixed and paraffin-embedded samples were derived from untreated patients. Sections from each patient were stained for 12′ at 37 °C with polyclonal rabbit anti-human DR4 (1:50; ab8415), anti-human DR5 (1:100; ab8416) and anti-human Osteoprotegerin (OPG) (1:200; ab73400; all from Abcam) after retrieval in citrate buffer (pH 7,6), 60′ for DR4 and DR5 and 30′ for OPG. DAB Detection Kit (Ventana) was introduced as antibody binding detector. Sections were examined by Zeiss Axiovert 200 M (Zeiss) and photomicrographs were acquired by AxioCam HRC camera and Axiovision Rel. 4.8 software (Zeiss).

### Statistical analyses

Data have been analysed using Microsoft Excel 2010 and are expressed as mean values ± standard error of the mean (SEM) and unpaired 2-tailed Student’s t-test was used considering p ≤ 0.05 as statistically significant. Anova test was performed using GraphPad Prism software, considering p ≤ 0.05 as statistically significant.

## Supplementary information

Supplementary information

## Data Availability

All data generated or analyzed during this study are included in this published article and its supplementary information files.
