# Supplementary material for: Soluble TRAIL Armed Human MSC As Gene Therapy For Pancreatic Cancer
Source: Sci Rep. 2019 Feb 11;9:1788. doi: 10.1038/s41598-018-37433-6 (PMC6370785; doi:10.1038/s41598-018-37433-6)

Soluble TRAIL Armed Human MSC As Gene Therapy For Pancreatic Cancer

Carlotta Spano, Giulia Grisendi, Giulia Golinelli, Filippo Rossignoli, Malvina Prapa, Marco Bestagno, Olivia Candini, Tiziana Petrachi, Alessandra Recchia, Francesca Miselli, Giulia Rovesti, Giulia Orsi, Antonino Maiorana, Paola Manni, Elena Veronesi, Maria Serena Piccinno, Alba Murgia, Massimo Pinelli, Edwin M. Horwitz, Stefano Cascinu, Pierfranco Conte & Massimo Dominici

SUPPLEMENTARY MATERIALS AND METHODS

FACS Analyses

Primary human pancreatic cancer cells PK59 EPI were characterized for EpCAM expression by staining with Anti-human EpCAM (eBioscience Inc., San Diego, CA, USA) and IgG1 PE isotype control (BD Pharmingen, San Diego, CA, USA) (Supplementary Fig. S8a). Samples were analysed with FACS AriaIII (Becton Dickinson, Franklin Lakes, NJ). Collected data were elaborated by FACS Diva software (Becton Dickinson, Franklin Lakes, NJ). Immunophenotype of the ex vivo expanded AD-MSC was performed by FACS analyses. Both not infected and lentiviral infected (sTRAIL and EV control) AD-MSC were analysed. The following panel of monoclonal antibodies was introduced: HLA-DR-FITC, CD14-PE, CD45-APC (all from BD Biosciences, San Jose, CA, USA), CD105-FITC, CD73-PE (BD Pharmingen, San Diego, CA, USA) and CD90-APC (eBioscience Inc., San Diego, CA, USA). FITC, PE (BD Pharmingen, San Diego, CA, USA) and APC (Miltenyi Biotec) appropriate isotype controls were used (Supplementary Fig. S1).

ELISA

ELISA test has been performed to detect secretion of Osteoprotegerin (OPG), a soluble decoy receptor of TRAIL, by PDAC cells (BxPC-3, MIA PaCa-2 and PK59 EPI) using Osteoprotegerin Human ELISA Kit (Abcam, Cambridge, UK) following manufacturer’s instructions. The PDAC culture media were replaced with fresh media when cell were sub-confluent and supernatants were collected (from confluent plates) after 24 hours of culture (Supplementary Fig. S3).

Differentiation Assays

The differentiation potential of expanded and transduced AD-MSC (Supplementary Fig. S2) was assessed as follows. MSC were seeded in culture medium until confluence and bone induction was started by α-MEM (GIBCO, Thermoscientific, Waltham, MA, USA) supplemented with 2,5 human platelet lysates (PL; from the Policlinic of Modena Blood Bank, Modena, Italy), 1% of penicillin/streptomycin (Carlo Erba Reagents Srl, Cornaredo, Italy) (P/S; 10^4 UI/ml and 10 mg/ml), Glutamine (BioWhittaker, Lonza, Verviers, Belgium) (2mM) and supplemented with dexamethasone (10 nM), L-ascorbic acid-2-phosphate (0,1 mM), beta-glycerol phosphate (10 mM) (all by Sigma Aldrich, St. Louis, MO, USA). For the second week the medium was additionally supplemented with BMP-2 (100 ng/ml) (Peprotech Inc., Rocky Hill, NJ, USA) was introduced. After 2 weeks of induction, differentiated MSC and controls were stained by Von Kossa stain: cells were fixed on ice cold methanol for 2 minutes, rinsed in distilled water and incubated with 1% silver nitrate (v/v; Sigma) for 30 minutes under a UV lamp. Stained samples were then washed and visualized by 10X magnification using an inverted microscope (Axio Observer A1 with color Axiocam MRC5 and Axiovision 4.82 software; Zeiss). MSC were induced toward the adipogenic lineage using DMEM low glucose **(**Euroclone SpA, Milan, Italy) additioned with 1% P/S, 10% rabbit serum (Euroclone), 5% Horse Serum (Hyclone) supplemented with dexamethasone (1µM), indomethacin (60 µM), rh-insulin (10 µM) and isobutylmethylxantine (IBMX) (0.5 mM) (all from Sigma). MSC were maintained in differentiation medium for 10 days replaced every 3 days and visualization of adipocyte differentiation was performed with Oil-Red-O solution (Sigma) and by microscopical observation (Axiovert 200, Zeiss). Adipogenic differentiation was evaluated by appearance of characteristic clusters of cells containing lipid vacuoles, stained in red. To test the chondrogenic differentiation MSC were plated in 15 ml conical tubes ($2x10^5$ cells/ml) in DMEM high-Glucose (GIBCO, Thermoscientific, Waltham, MA, USA) supplemented with bone morphogenic protein-6 (500 ng/ml; PeproTech), transforming growing factor-β (10 ng/ml; PeproTech), ITS+Premix 50 mg/ml (containing insulin, 6.25 µg/ml; transferrin, 6.25 µg/ml; selenous acid, 6.25 ng/ml; bovine serum albumin, 1.25 mg/ml; linoleic acid, 5.35 µg/ml; BD Biosciences), dexamethasone final concentration (100 nM), L-ascorbic acid-2-phosphate (0,2 mM), sodium pyruvate (100X, 100 µg/ml), proline (40

μg/ml), glutamine (100X, 100 nM) and Pen/Strep 1% (all from Sigma). The cells were centrifuged to the bottom of a 15 ml conical tube and kept in incubator with controlled atmosphere (5% CO_2 and temperature of 37°C); the medium was changed every 2 days, leaving the pellet undisturbed inside the tube. At 21 days of differentiation, the pellets were harvested, formalin fixed and paraffin embedded. Serial sections of induced and uninduced samples were then specifically stained with the Alcian blue solution (1%).

Dose response assay

BxPC-3 cells were seeded in 96-well tissue culture plates. After 12h, cells were treated with increasing doses (from 10 to 20.000 ng/ml) of rhTRAIL (Peprotech) for another 24h. Cytotoxicity was assessed by MTS assay (CellTiter 96® AQueous Assay System, Promega, Wisconsin, USA) according to the manufacturer's instructions (Supplementary Fig. 4).

Apoptosis Assay

In order to assess the anti-angiogenic effect of sTRAIL released by transduced AD-MSC, the percentage of HUVEC death was measured by Propidium Iodide staining (Supplementary Fig. 7b). HUVEC were seeded ($2x10^4$/well in 12 well plate) and the day after the AD-MSC supernatants were added to the culture. Unlike for the cytotoxicity studies against PDAC cell lines, supernatants were collected culturing gene modified AD-MSC with HUVEC medium (M-200PRF plus LSGS Kit; GIBCO) for 48 hours. Control medium (CTL) alone and rhTRAIL (20 μg/ml) diluted into M-200PRF/ LSGS medium were used as controls.

Immunohistochemistry and immunocytochemistry

Formalin-fixed, paraffin-embedded liver sections from mice of the in vivo study were stained by hematoxylin-and-eosin (Sigma-Aldrich, St Louis, MO) and visualized by microscopy to exclude treatment-related alterations of liver (Supplementary Figure 6b).

In order to quantify the number of intra-tumoral vessels (Supplementary Figure 7a), formalin-fixed, paraffin-embedded tumor sections were retrieved in citrate buffer (pH 6) for 15 minutes and incubated overnight at 4°C with the following primary antibody: rabbit polyclonal anti CD31 (1:50; ab28364. Abcam, Cambrige, UK). Slides were then incubated by a biotinylated goat anti-rabbit IgG (H+L) (1:200; Vector Laboratories, Burlingame, CA) for 1 hour at room temperature. Negative controls were run simultaneously omitting primary antibody. Staining was performed and visualized by 3.3 0-diaminobenzidine (DAB) (in brown, Vector Laboratories). All slides were counterstained with Harris hematoxylin (Bio Optica, Milan, Italy). CD31 positive vessels number was evaluated in 10 field/sections for each tumor (magnification: 10X).

Human primary pancreatic cancer cells PK59 EPI were maintained in culture and detached using Trypsin EDTA 1X in PBS (Euroclone SpA, Milan, Italy). After one wash with PBS $1x10^5$ cells were resuspended in 200 µl of cold PBS and spotted by Shandon Cytospin 4 Cytocentrifuge (Thermoscientific, Rockford, Illinois, USA) at 450 rpm for 15’. Spots were then fixed in cold methanol (Carlo Erba, Val De Reuil, France) for 15’ and subsequently dried at room temperature overnight before proceeding with the staining. Spots were stained with hematoxylin-and-eosin (Carlo Erba; Supplementary Figure S8b). Moreover, in order to evaluate cytokeratin-7 (CK-7) expression, we performed a staining using an anti-CK-7 antibody, SP52 (undiluted, Ventana) and the biotinylated goat anti-rabbit IgG (H+L) (1:200; Vector Laboratories, Burlingame, CA) for 1 hour at room temperature each. Negative control was run simultaneously by omitting primary antibody. All slides were counterstained with Harris hematoxylin (Bio Optica, Milan, Italy). Sections were examined by Zeiss Axio Observer Z.1 (Zeiss, Oberkochen, Germany) and photomicrographs were acquired by AxioCam 506 color camera and ZEN software (Zeiss).

SUPPLEMENTARY FIGURE LEGENDS

Supplementary Figure S1. AD-MSC maintain their immunophenotype after lentiviral infection. FACS analysis of wild type and transduced AD-MSC: a representative dot plot illustrates physical parameters of AD-MSC (upper left panel; forward Scatter, FSC, and side scatter, SSC). Proper isotype controls have been used for each fluorochrome (first row of plots). Both empty vector and soluble TRAIL AD-MSC lack the expression of HLA-DR, CD14 and CD14 while they are positive for CD105, CD73 and CD90, as wild type AD-MSC.

Supplementary Figure S2. AD-MSC maintain their capacity to differentiate after gene modification. Representative photomicrographs showing adipogenic (left, Oil red O staining, magnification 400X), osteogenic (middle, Von Kossa staining, magnification 10X) and chondrogenic (right, Alcian Blue staining, magnification 20X) differentiations of WT, EV and sTRAIL producing AD-MSC.

Supplementary Figure S3. Target pancreatic cancer cell lines and primary cancer cells secrete different amounts of OPG. Histogram representing different secretion levels (expressed as pg/ml ±SEM) measured by ELISA of the target pancreatic cancer cells. BxPC-3 cell line produced 355.65±8.7 pg/ml in 24 hours, while MIA PaCa-2 secreted 3.89 (±0.73). Primary human pancreatic cancer cells PK59 released 16512.2 (±0.74) in the same interval of time.

Supplementary Figure S4. BxPC-3 PDAC cell line is sensitive at rhTRAIL in a dose-dependent manner. BxPC-3 sensitivity to rhTRAIL depends on the concentration of the pro-apoptotic agent into the culture medium. PDAC cells start to die after 200 ng/ml and reach the plateau between 500 and 1000 ng/ml of rhTRAIL.

Supplementary Figure S5. Freezing (-80°C) of AD-MSC supernatants does not negatively impact on apoptotic activity of sTRAIL. Histogram of propidium Iodide staining by FACS showing the mortality of BxPC-3 cultured for 24 hours with either empty vector (EV) or soluble TRAIL (sTRAIL) AD-MSC supernatants used both as fresh and frozen products. No statistically significant differences ($p>0.05$) were observed inside the groups comparing fresh versus frozen.

Supplementary Figure S6. In vivo treatment with AD-MSC armed with sTRAIL does not impair animal health. During in vivo experiments, NOD/SCID mice overall status was routinely checked, with particular attention for behaviour, food intake and weight. **a.** As showed in the graph, animal weight within different groups compared did not change appreciably ($p\geq0.2$) during all the experiment duration (38 days). **b.** Hematoxylin & eosin staining of mice liver sections (4μm) did not reveal pathological features in the different groups.

Supplementary Figure S7. Impact of AD-MSC TRAIL on angiogenesis. a. In vivo CD31 vessels count in tumour samples isolated from different animal groups (CTL; EV; rhTRAIL, sTRAIL). Vessel density is significantly reduced after sTRAIL treatment. **b.** In vitro quantification of HUVEC cell death at 24 hours by PI staining and FACS analysis. Conditioned media from AD-PC sTRAIL significantly increase HUVEC cell mortality compared to EV or rhTRAIL (1ug/ml).

Supplementary Figure S8. Primary human cells PK59 EPI expresses typical PDAC extracellular antigens. a. FACS analysis of PK59 EPI PDAC primary cells stained with anti-Epithelial Cell Adhesion Molecule (EpCAM) antibody showing that the population homogeneously expresses high levels of this marker. **b.** Hematoxylin and Eosin (H&E) and immunocytochemical staining of PK59 EPI PDAC cells using anti-CK-7 antibody. Isotype staining was performed in parallel

as control. The staining shows an intense positivity of the cellular population (in brown). Magnification 100X, scale bar 100 μm.

SUPPLEMENTARY FIGURES

Supplementary Figure S1. AD-MSC maintain their immunophenotype after lentiviral infection.

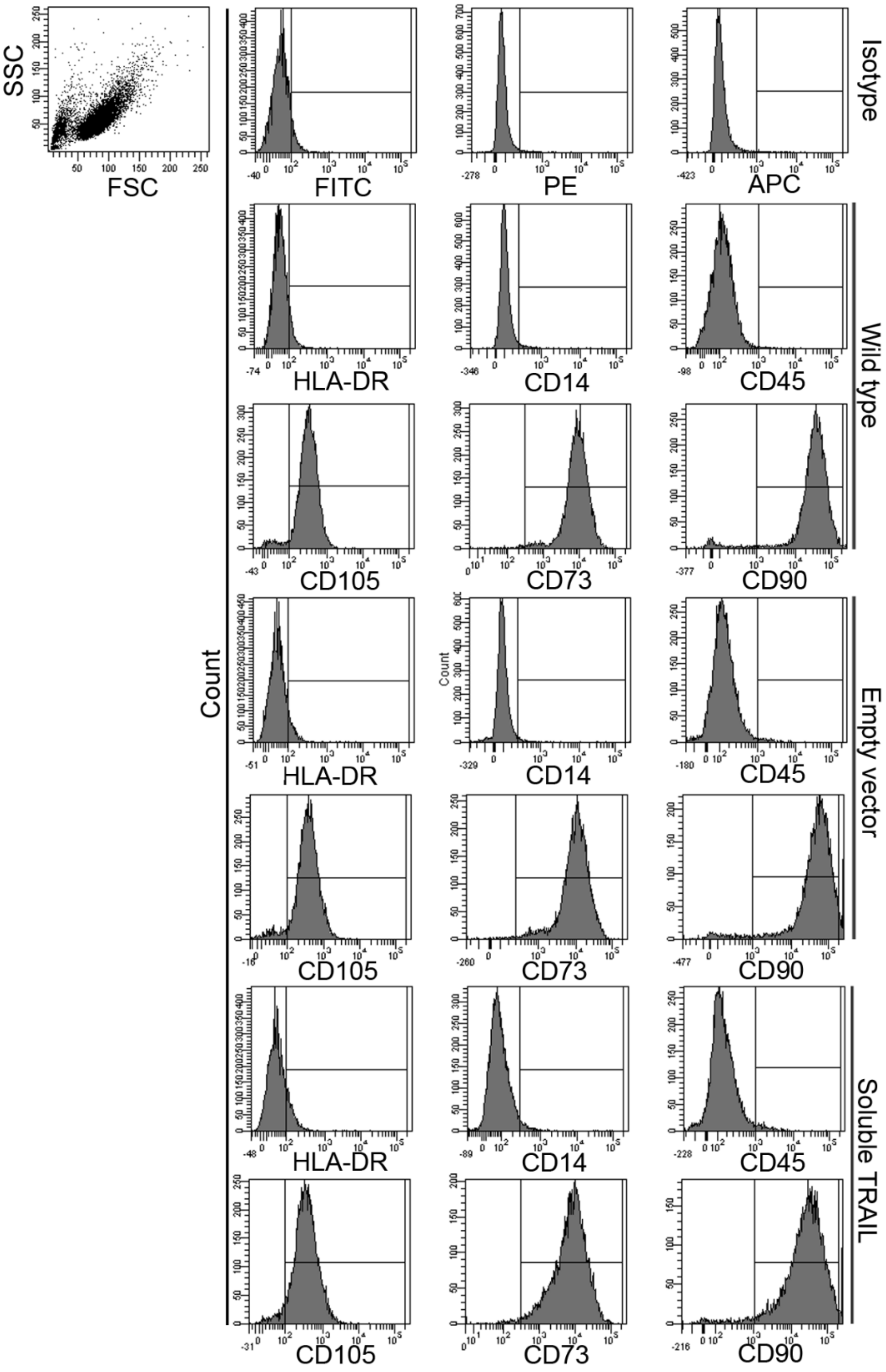

Supplementary Figure S2. AD-MSC maintain their capacity to differentiate after gene modification.

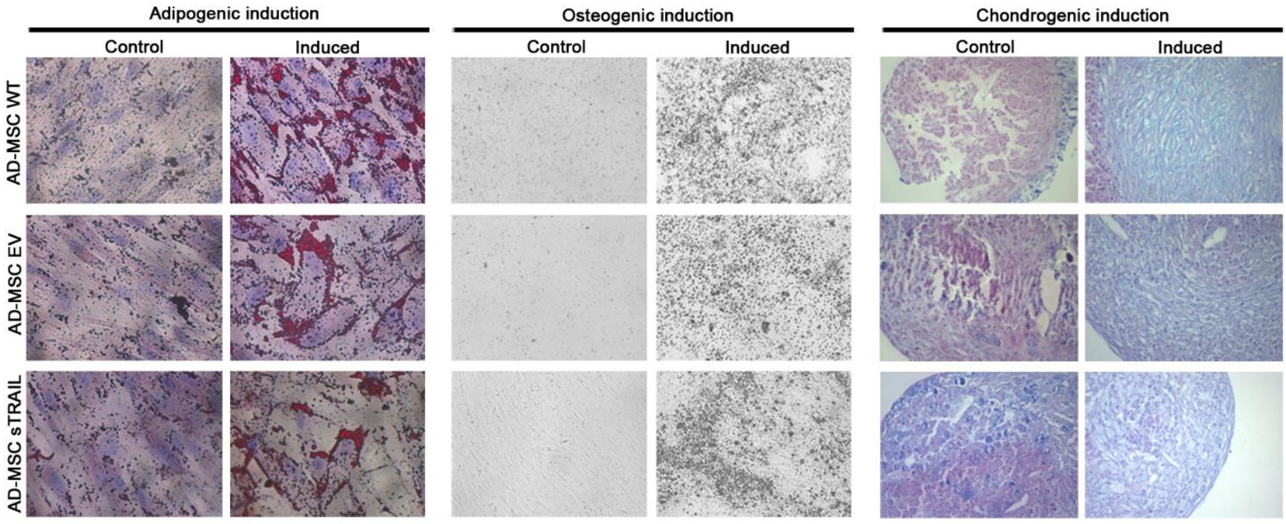

Supplementary Figure S3. Target pancreatic cancer cell lines and primary cancer cells secrete different amounts of OPG.

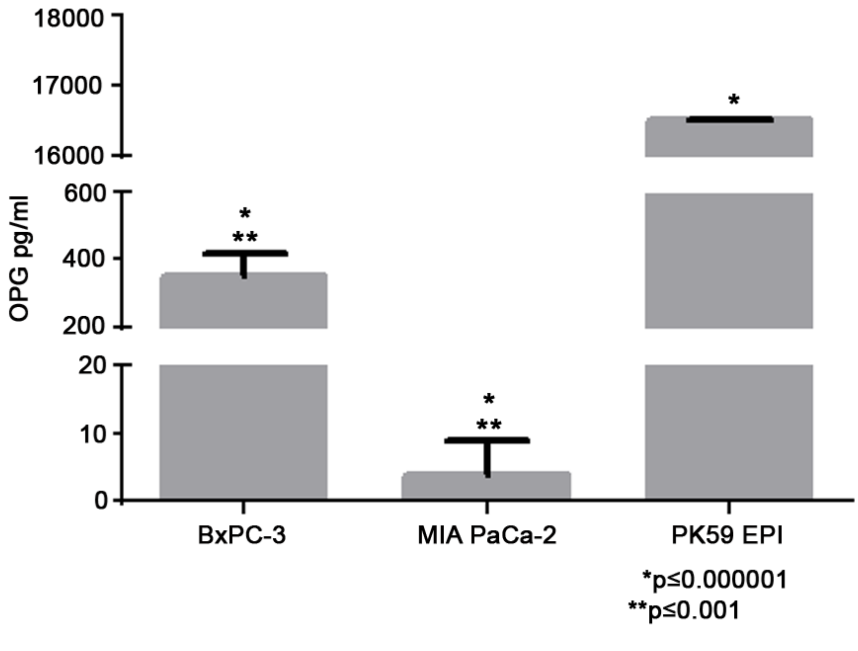

Supplementary Figure S4. BxPC-3 PDAC cell line is sensitive at rhTRAIL in a dose-dependent manner.

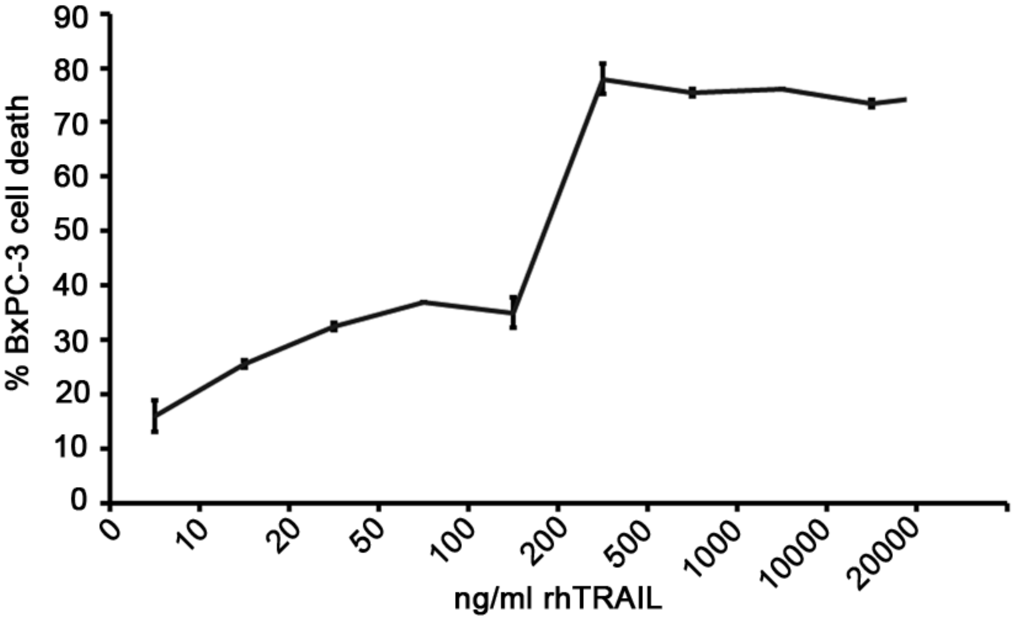

Supplementary Figure S5. Freezing (-80°C) of AD-MSC supernatants does not negatively impact on apoptotic activity of sTRAIL.

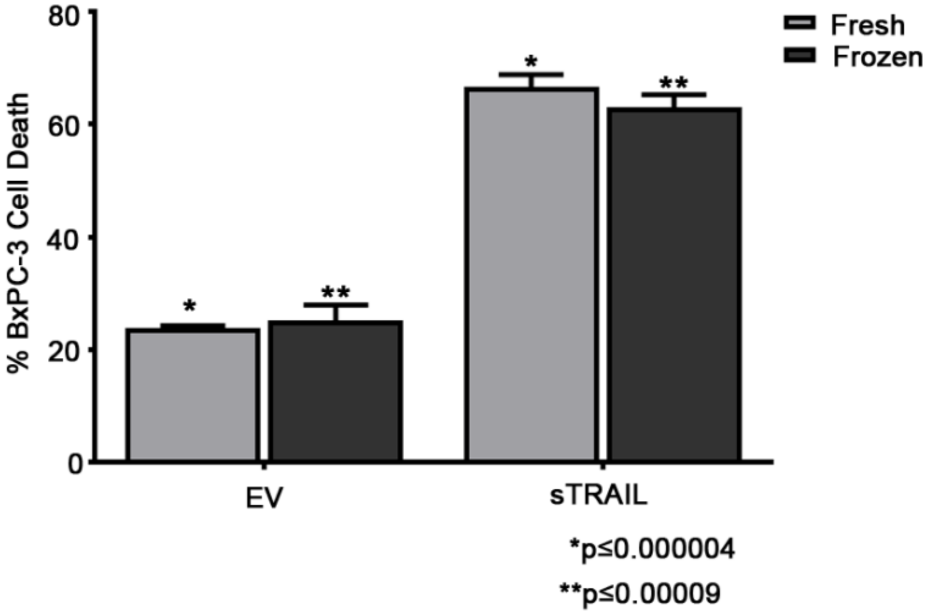

Supplementary Figure S6. In vivo treatment with AD-MSC armed with sTRAIL does not impair animal health.

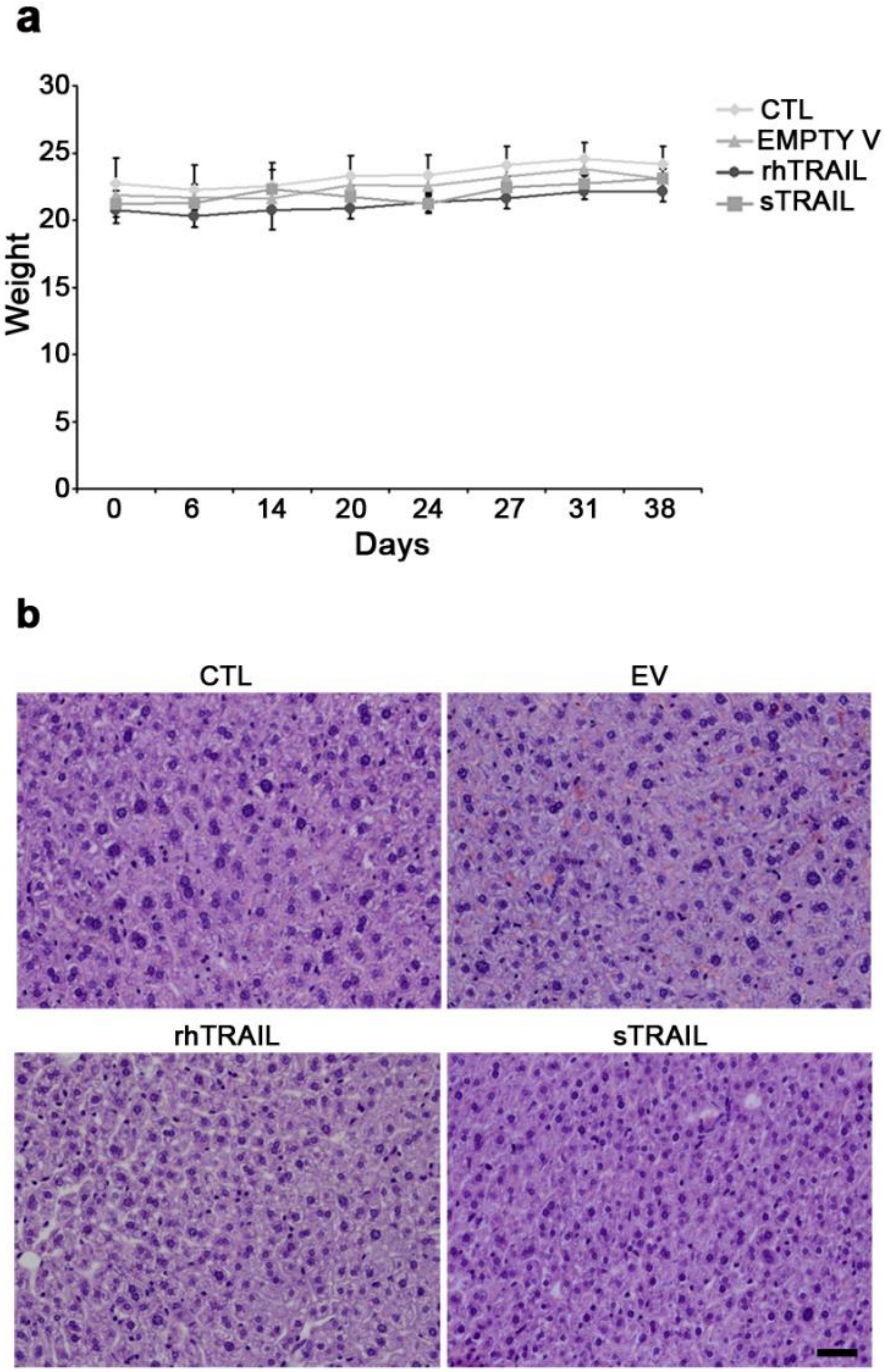

Supplementary Figure S7. Impact of AD-MSC TRAIL on angiogenesis.

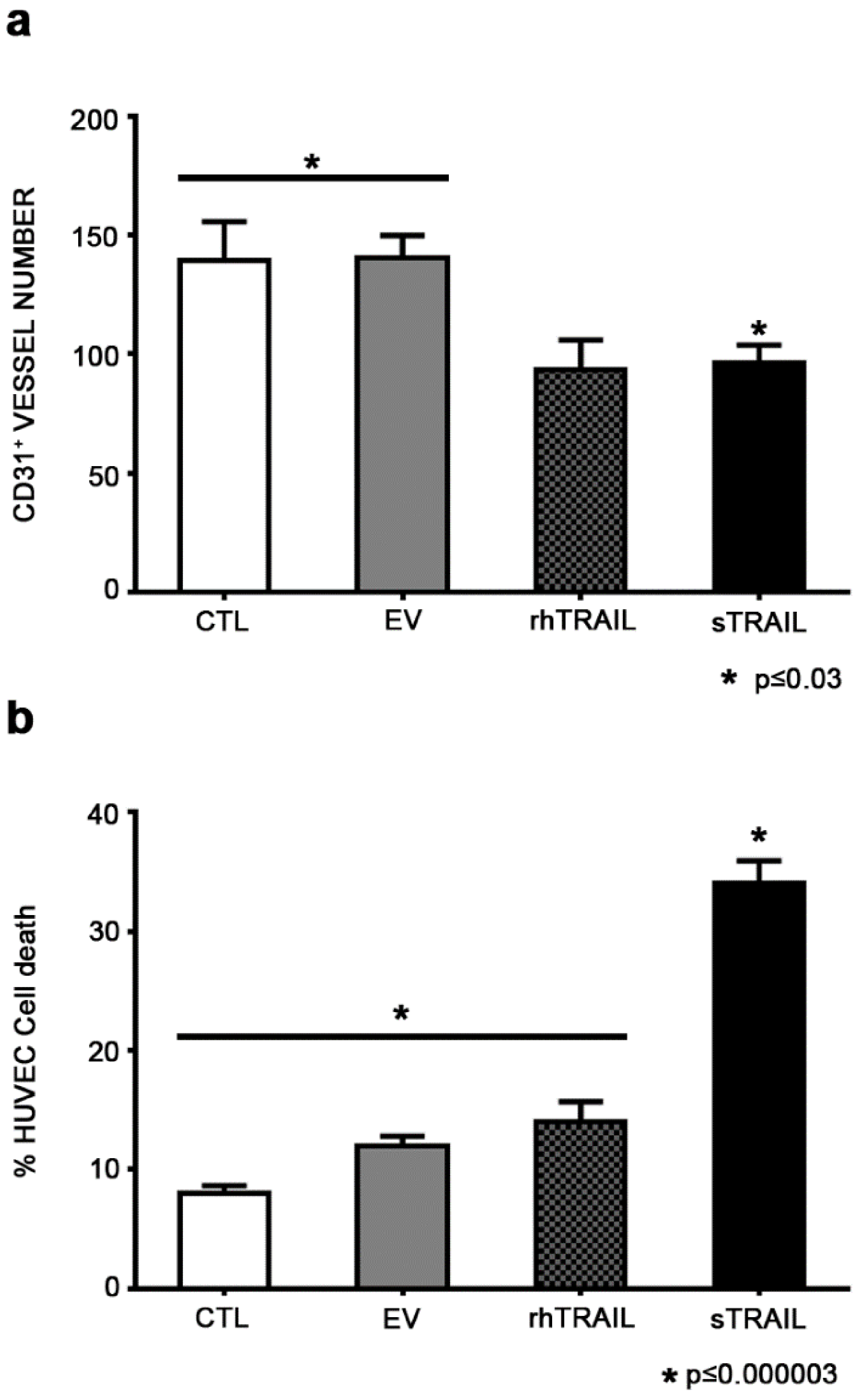

Supplementary Figure S8. Primary human cells PK59 EPI expresses typical PDAC extracellular antigens.

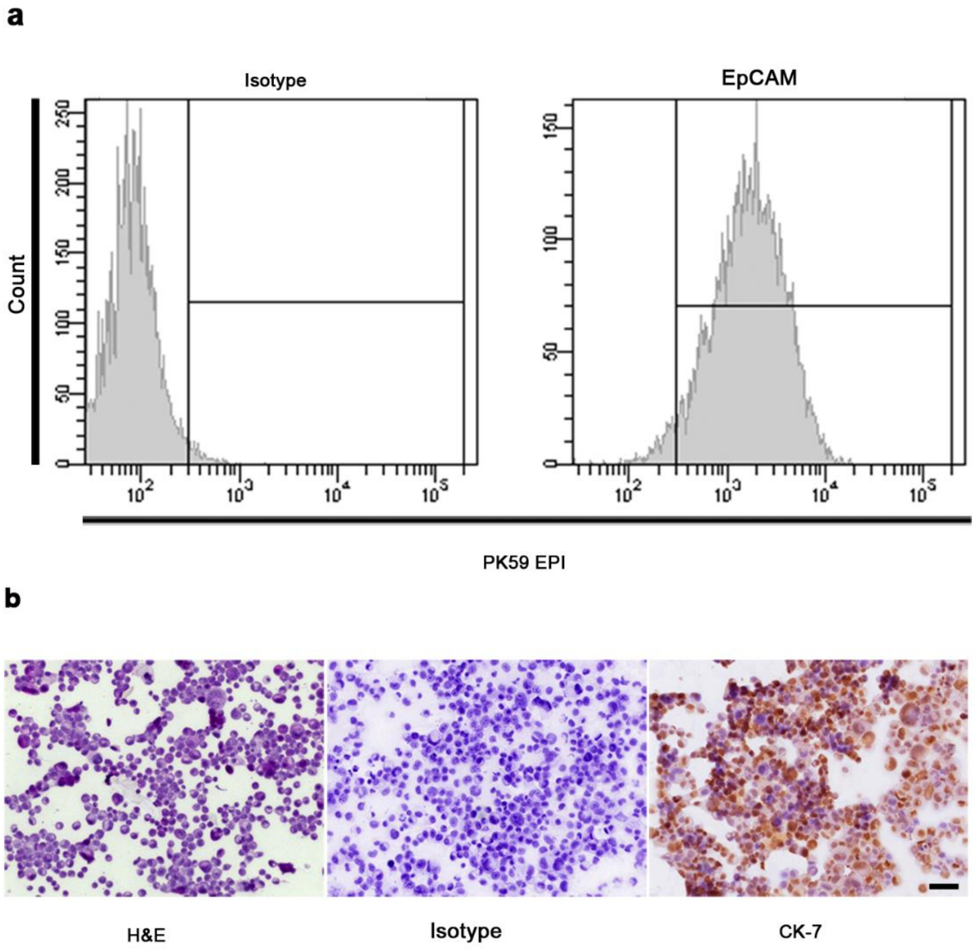

Supplementary Figure S9: Gels/Blots (Expanded data)

Figure 1b

Full length agarose gel

WPRE, Grey scale (Exported .tiff image from ChemiDoc XRS+, Biorad)

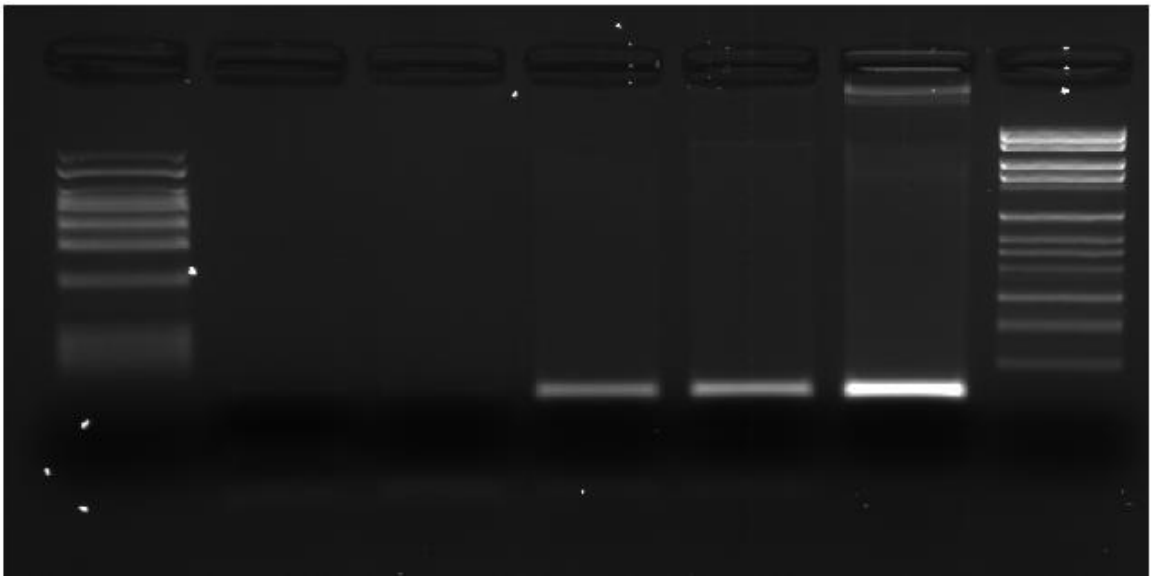

WPRE, Grey scale inverted

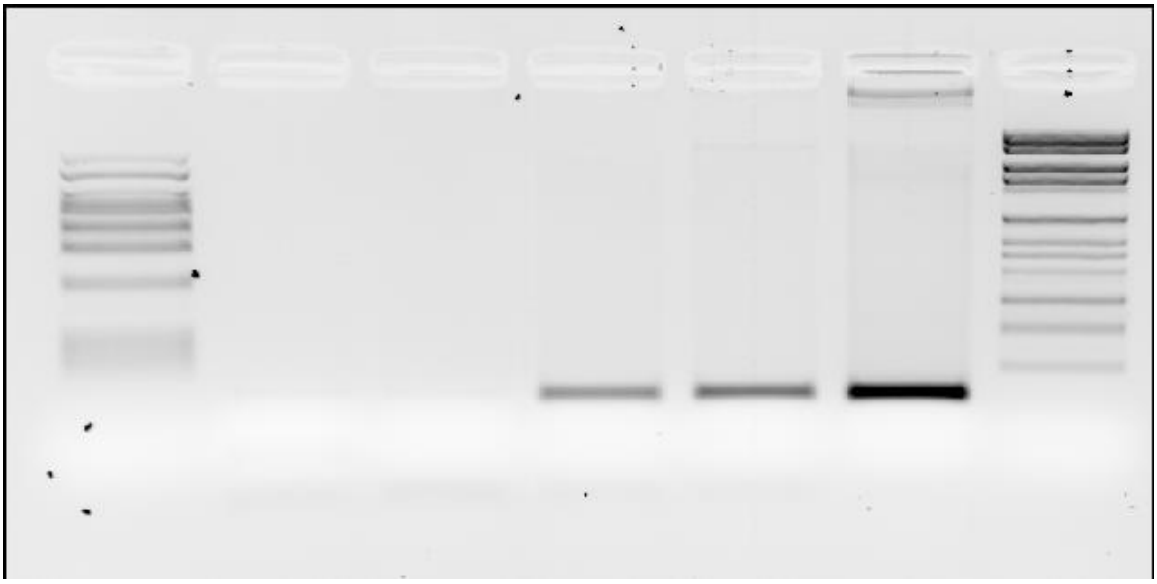

Figure 1b

Full length agarose gel

Human β-Actin, Grey scale (Exported tiff image from Gel Doc, ChemiDoc XRS+, Biorad)

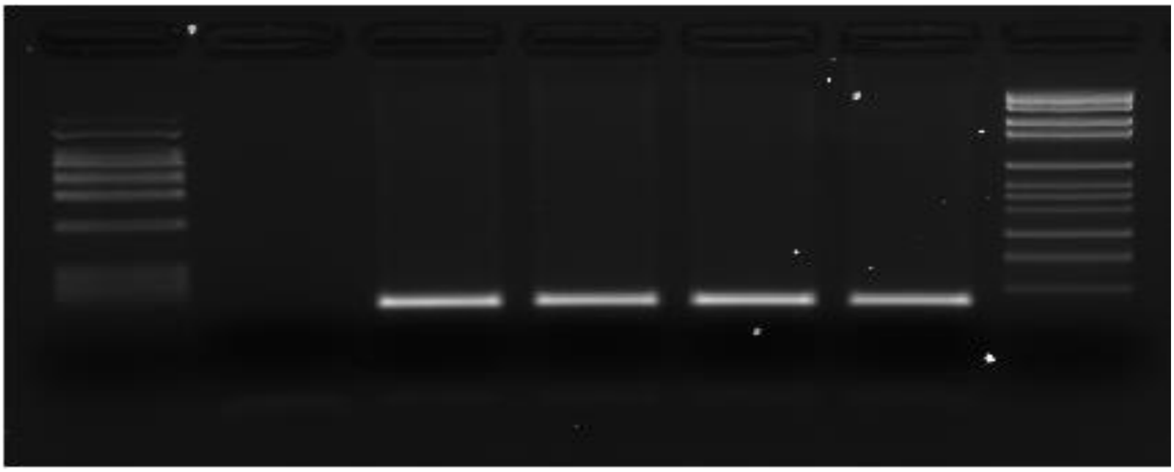

Human β-Actin, Grey scale inverted

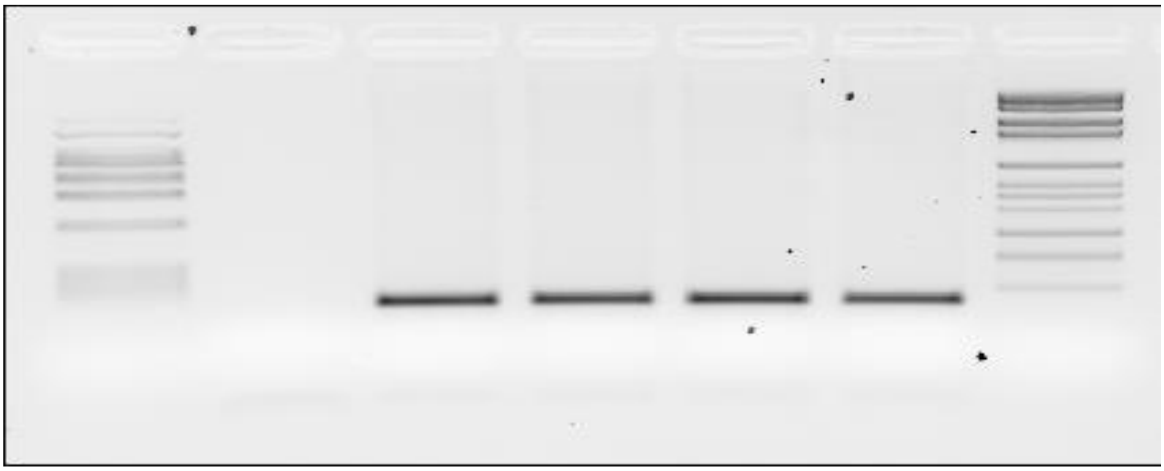

Figure 1e

Full length Western blot

sTRAIL trimer detection (jpeg image from Odissey LI-COR)

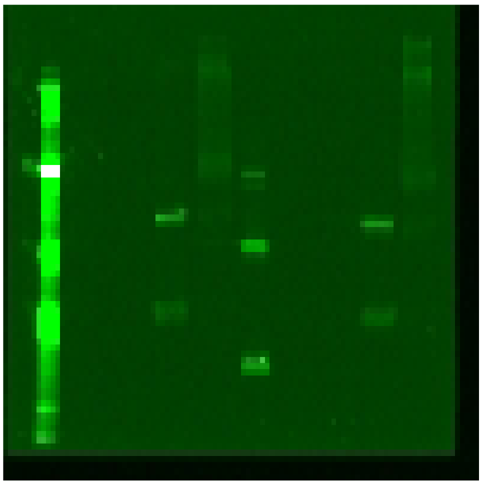

sTRAIL trimer detection, Grey scale inverted with modified brightness/contrast

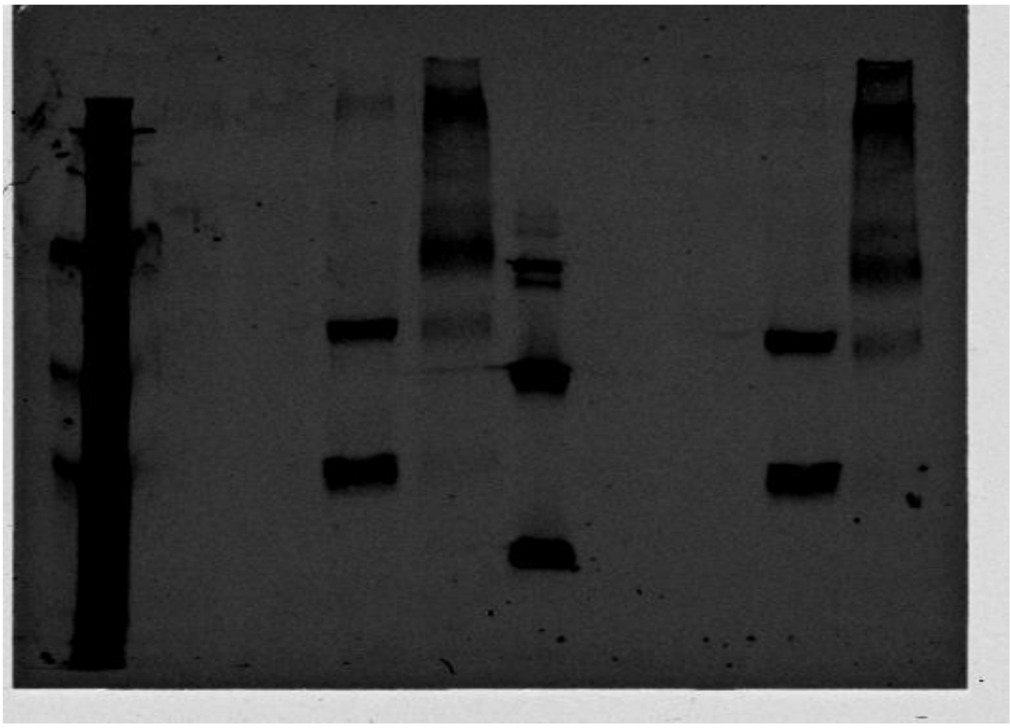

Figure 2c

Full length Western blot

Caspase 8 detection (jpeg image from Odissey LI-COR)

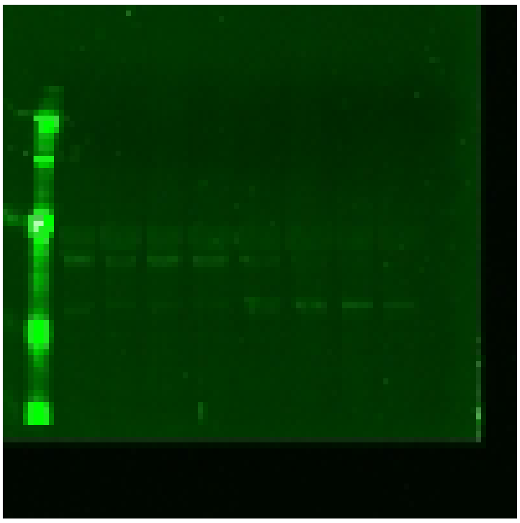

Caspase 8 detection, Grey scale inverted with modified brightness/contras

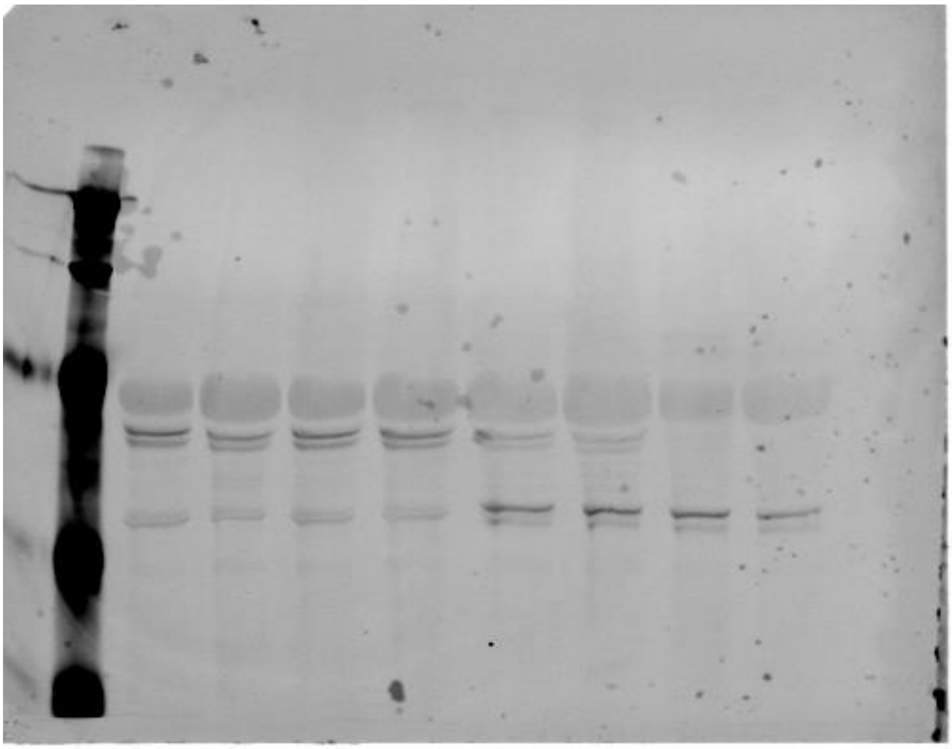

Figure 2c

Full length Western blot

GAPDH detection (jpeg image from Odissey LI-COR)

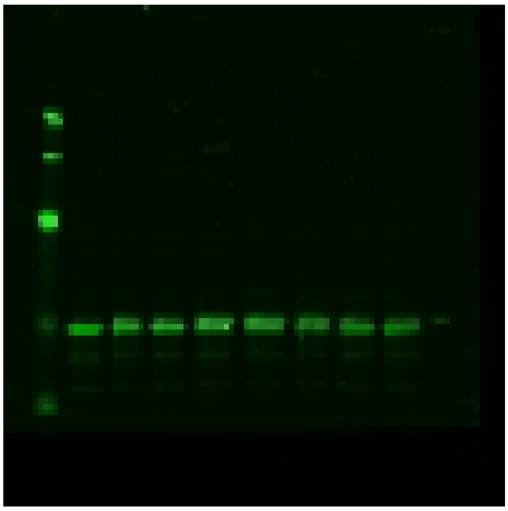

GAPDH detection, Grey scale inverted modified brightness/contrast

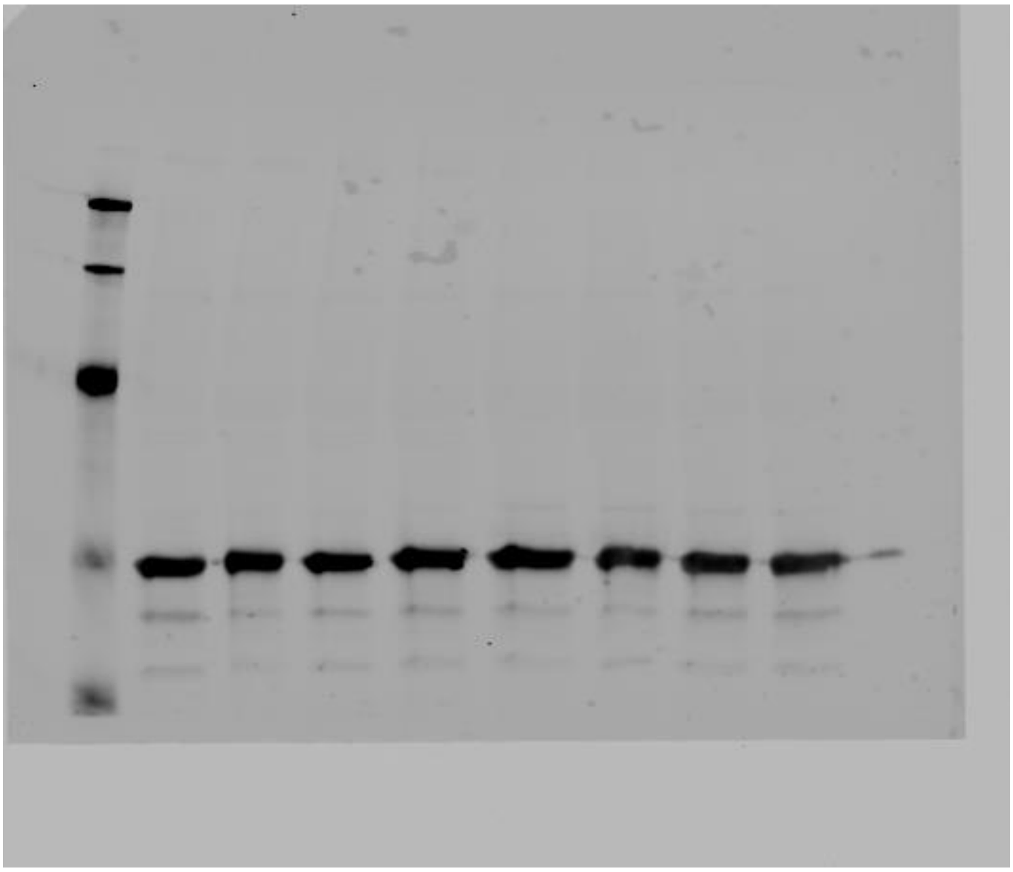

Supplement: Supplementary file 1 — Supplementary information [file 41598_2018_37433_MOESM1_ESM.pdf]
